# HIV Prevention and Care Among Black Cisgender Sexual Minority Men and Transgender Women: Protocol for an HIV Status–Neutral Cohort Study Using an Observational-Implementation Hybrid Approach

**DOI:** 10.2196/48548

**Published:** 2023-12-01

**Authors:** Justin R Knox, Brett Dolotina, Tyrone Moline, Isabella Matthews, Mainza Durrell, Hillary Hanson, Ellen Almirol, Anna Hotton, Jade Pagkas-Bather, Yen-Tyng Chen, Devin English, Jennifer Manuzak, Joseph E Rower, Caleb Miles, Brett Millar, Girardin Jean-Louis, H Jonathon Rendina, Silvia S Martins, Christian Grov, Deborah S Hasin, Adam W Carrico, Steve Shoptaw, John A Schneider, Dustin T Duncan

**Affiliations:** 1 Mailman School of Public Health Columbia University New York, NY United States; 2 Chicago Center for HIV Elimination University of Chicago Chicago, IL United States; 3 Department of Psychiatry Rutgers University New Brunswick, NJ United States; 4 Division of Immunology School of Medicine Tulane University New Orleans, LA United States; 5 Center for Human Toxicology University of Utah Salt Lake City, UT United States; 6 Department of Psychiatry and Behavioral Sciences Miller School of Medicine University of Miami Coral Gables, FL United States; 7 Department of Epidemiology Milken Institute School of Public Health George Washington University Washington, DC United States; 8 Einstein-CUNY-Rockefeller Center for AIDS Research School of Public Health and Health Policy City University of New York New York, NY United States; 9 Department of Family Medicine David Geffen School of Medicine University of California, Los Angeles Los Angeles, CA United States

**Keywords:** HIV, cisgender sexual minority men, transgender women, Black, African American, cannabis, sleep, stimulants, substance use, alcohol

## Abstract

**Background:**

Black cisgender gay, bisexual, and other sexual minority men (SMM) and transgender women (TW) continue to be heavily affected by HIV. Further research is needed to better understand HIV prevention and care outcomes in this population. In particular, there is a need for research examining the impact of substance use and sleep health on HIV prevention and treatment outcomes among Black SMM and TW.

**Objective:**

This paper outlines the study methods being used in the recently launched follow-up study to the Neighborhoods and Networks (N2) study, which we refer to as N2 Part 2 (N2P2). N2P2 aims to address this gap in the literature, build off the findings of the original N2 study, and identify socioenvironmental determinants of health, including whether neighborhood and network factors mediate and moderate these relationships.

**Methods:**

Building on the N2 cohort study in Chicago from 2018 to 2022, N2P2 used a prospective longitudinal cohort design and an observational-implementation hybrid approach. With sustained high levels of community engagement, we aim to recruit a new sample of 600 Black SMM and TW participants residing in the Chicago metropolitan statistical area. Participants are asked to participate in 3 study visits across an 18-month study period (1 visit every 9 months). Four different forms of data are collected per wave: (1) an in-person survey, (2) biological specimen collection, (3) a daily remote ecological momentary assessment for 14 days after each study visit, and (4) data from electronic health records. These forms of data collection continue to assess neighborhood and network factors and specifically explore substance use, sleep, immune function, obesity, and the implementation of potential interventions that address relevant constructs (eg, alcohol use and pre-exposure prophylaxis adherence).

**Results:**

The N2P2 study was funded in August 2021 by the National Institute of Drug Abuse (R01DA054553 and R21DA053156) and National Heart, Lung, and Blood Institute (R01HL160325). This study was launched in November 2022. Recruitment and enrollment for the first wave of data collection are currently ongoing.

**Conclusions:**

The N2P2 study is applying innovative methods to comprehensively explore the impacts of substance use and sleep health on HIV-related outcomes among an HIV status–neutral cohort of Black SMM and TW in Chicago. This study is applying an observational-implementation hybrid design to help us achieve findings that support rapid translation, a critical priority among populations such as Black SMM and TW that experience long-standing inequities with regard to HIV and other health-related outcomes. N2P2 will directly build off the findings that have resulted from the original N2 study among Black SMM and TW in Chicago. These findings provide a better understanding of multilevel (eg, individual, network, and neighborhood) factors that contribute to HIV-related outcomes and viral suppression among Black SMM and TW.

**International Registered Report Identifier (IRRID):**

DERR1-10.2196/48548

## Introduction

### Background

Black cisgender gay, bisexual, and other sexual minority men (SMM) and transgender women (TW) continue to be heavily affected by HIV, with both groups at increased vulnerability for HIV acquisition [1-8]. Despite accounting for <1% of the US population, Black SMM constituted 28% of newly diagnosed HIV cases in 2020 [1]. Black TW also experience disparately high rates of HIV prevalence, with a recent systematic review estimating HIV prevalence to be 14.1% among TW, overall, and 44.2% among Black TW, in particular [9]. Despite these long-standing inequities, much of the research on HIV among SMM and TW has been conducted among racially and ethnically diverse samples, and, in some instances, focused on comparing Black and White populations. In contrast, this study focuses exclusively on Black SMM and TW as a more intentional, effective, and efficient research approach that recognizes the nuanced challenges faced by these communities.

Within this context, intersectionality theory provides a framework for understanding and examining the ways in which intersecting societal oppressions have multiplicative effects on health outcomes among multiply marginalized people. Indeed, in a society structured by White supremacy, cisnormativity, and heteronormativity, Black SMM and TW experience multilevel, interrelated oppressions that directly impact their life experiences, including factors related to HIV prevention and care [10-13]. It is important to recognize that Black SMM and TW experience these oppressions in distinct ways [14]. However, in certain local contexts (eg, Chicago), there is also a significant overlap of social and sexual networks as well as neighborhood-related factors between Black SMM and TW [15-17]. Given the long-standing health inequities experienced by Black SMM and TW [4,9,18,19], such as those related to HIV prevention, care, and its determinants, research to further understand salient issues in the lives of these multiply marginalized populations is urgently needed.

Current research on HIV prevention and care should be responsive to a dynamic biomedical landscape. Specifically, HIV pre-exposure prophylaxis (PrEP) represents an unrealized yet potentially critical tool for ending the HIV epidemic (EHE) [20] and reducing inequities [21-28]. Although PrEP awareness and use have been increasing [29-32], there has been limited uptake of PrEP among marginalized populations, especially Black SMM and TW [33-38]. For example, in 2020, among those for whom PrEP is recommended, only 9% of Black individuals were on PrEP (as opposed to 66% of White individuals) [39]. Similar levels of suboptimal PrEP uptake have been noted in TW populations as well [26]. Antiretroviral therapy (ART) is another critical tool for EHE as it functions to maintain undetectable levels of HIV viral load among people living with HIV [40]. Achieving sustainable levels of effective ART adherence is critical for HIV prevention and treatment, as research demonstrates that viral load is associated with onward HIV transmission [41], such that no HIV transmission events occur when viral loads are below a certain threshold (eg, undetectable=untransmissible) [42]. However, evidence indicates that many Black SMM and TW face challenges in achieving sustained viral suppression, including factors such as stigmatizing experiences, depression, anxiety, intimate partner violence, and financial stressors [43-48].

Further research is needed to better understand HIV prevention and care outcomes among Black SMM and TW, including the impact of prevalent behaviors such as substance use. Recent findings indicate that cannabis use, including heavy cannabis use, is highly prevalent among Black SMM and TW. For example, in the Neighborhoods and Networks (N2) study among Black SMM and TW in Chicago (mainly the south side of Chicago) [49], two-thirds of participants currently use cannabis (past month), half of whom use cannabis daily [50], similar to other studies among Black SMM and TW [48,51,52]. Cannabis use also likely increased during the COVID-19 pandemic [53,54], including for Black SMM and TW [50]. Thus far, however, there have been mixed findings as to whether cannabis use impacts HIV-related outcomes [55,56]; although among Black SMM and TW, studies have shown that cannabis use is associated with HIV acquisition [51], being connected to an HIV transmission cluster [57], and decreased HIV testing [58]. The impact of cannabis use on the PrEP care continuum has not been studied as much, although initial results suggest that there may be an association [55,59-61], including a few studies on Black SMM [59,60]. Additional substance use behaviors among Black SMM and TW (beyond cannabis use) are also potentially relevant to understanding HIV-related outcomes. Alcohol misuse, including heavy drinking and binge drinking, is prevalent and has been shown to negatively impact HIV-related outcomes, including in Black SMM and TW [62-64]. Increasing methamphetamine use among Black SMM and TW is also a growing concern [65,66], especially given its negative impact on HIV prevention [67,68], the acquisition of other sexually transmitted infections (STIs) [69-71], and HIV treatment outcomes [72,73].

There are critical gaps in our understanding of substance use among Black SMM and TW, including identifying its socioenvironmental determinants as well as its impacts on HIV-related outcomes. Further research into whether substance use impacts HIV-related outcomes should also focus on potential mechanisms (eg, biological vulnerability [74-79] and neurocognitive impacts [80-82]), which will help identify potential intervention points along causal pathways and enhance external validity, which can also help inform future intervention and implementation strategies [83]. When studying HIV prevention and care among Black SMM and TW, it is also critical to take contextual factors into account [84,85], particularly those operating at multiple socioecological levels [86]. Neighborhood and social factors have been shown to be associated with HIV-related outcomes in general populations as well as among intersectional minoritized populations, such as Black SMM and TW [87]. One especially important issue is policing and police brutality, as research has demonstrated that Black SMM and TW frequently encounter law enforcement [88,89], and the presence of police has been linked to be various HIV-related outcomes, including among Black SMM [90].

Sleep health is another salient factor in the lives of Black SMM and TW, as studies have found a high prevalence of sleep problems (eg, excessive or short sleep) [91,92], and this has worsened during the COVID-19 pandemic [93]. Sleep health has been found to be associated with HIV-related outcomes in the general population [94] as well as in samples of sexual and gender minority groups [95,96]. Research regarding the relationship between sleep and HIV outcomes among Black SMM and TW is scant, although 1 study from our team has demonstrated that poor sleep health is associated with lower PrEP adherence among Black SMM [93].

Generally, there have been limitations to the collective research that has been conducted, thus far, on substance use, sleep health, and their impacts on HIV-related outcomes among Black SMM and TW. First, most studies used cross-sectional data and relied on self-reporting (which are subject to social desirability bias). Studies that use objective measures, such as biological specimens and data from electronic health records (EHRs), allow researchers to assess the accuracy of self-report data and minimize self-report bias. Furthermore, most studies have used retrospective measures, which summarize across periods and are subject to recall bias [97]. Prospective data, especially granular data, can be collected using innovative methods such as ecological momentary assessment (EMA) [97,98], which captures day-to-day and event-level variation of experiential, spatial, and behavioral data in real time. Longitudinal studies that include objective, prospective, and granular measurement of relevant constructs and that evaluate mechanisms and context will allow us to better study these complex constructs and how they interrelate among Black SMM and TW.

### Objectives

**Figure 1:**
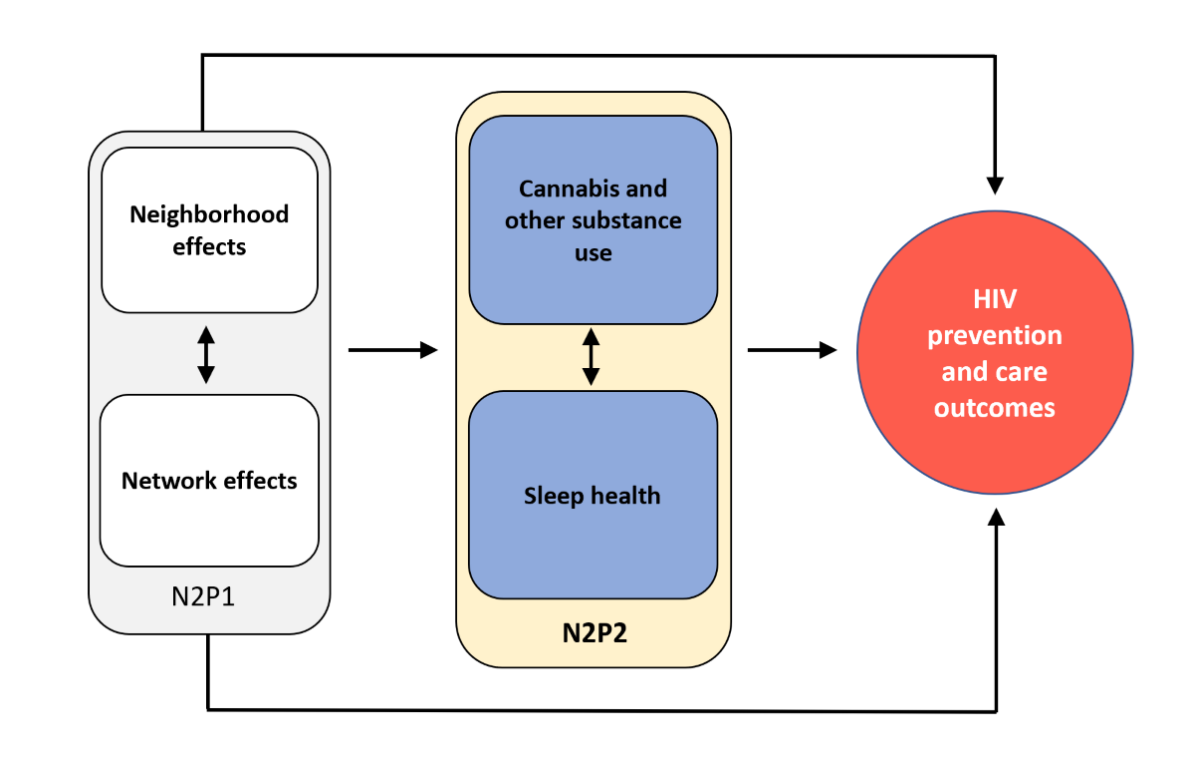
Conceptual model for the Neighborhoods and Networks Part 2 (N2P2) study. Originally looking at the neighborhood and network factors of HIV-related outcomes in the Neighborhoods and Networks Part 1 (N2P1) study, we have now launched the N2P2 study to expand our understanding of substance use, sleep, and HIV-related outcomes.

To overcome these limitations, we are conducting a prospective study to explore the impact of substance use and sleep health on HIV-related outcomes among a well-characterized HIV status–neutral cohort of Black SMM and TW in Chicago, Illinois. The study objectives are conceptualized in Figure 1, and the primary objectives are summarized as follows:

1. Determine the causal effects of substance use (eg, cannabis use and stimulant use) and sleep health on HIV outcomes, including acquisition, prevention (eg, HIV testing, PrEP uptake, and PrEP adherence), and care outcomes (eg, retention in care and viral suppression).
2. Assess whether associations between substance use, sleep health, and HIV prevention and care outcomes vary by contextual characteristics (eg, individual-, network-, and neighborhood-level constructs) and are mediated by relevant constructs (neurocognitive impacts and biological vulnerability).

To prioritize the potential expedited translation of study findings into public health impact, which is of critical importance among populations such as Black SMM and TW that experience long-standing health inequities, we will use an innovative observational-implementation hybrid approach [99]. The observational-implementation hybrid approach prioritizes the collection of implementation data while conducting observation research. This paper outlines the study methods being used in the recently launched follow-up study to the N2 study [49], which we refer to as N2 Part 2 (N2P2).

## Methods

### Study Design

Building on the N2 cohort study in Chicago, which was completed between January 2018 and June 2022 (N=412), the N2P2 study uses a prospective longitudinal cohort design and aims to recruit a sample of 600 Black SMM and TW participants residing in the Chicago metropolitan statistical area (MSA). Following updated recommendations for labeling hybrid studies [100], we will apply an observational-implementation hybrid approach in the context of the cohort study design [99]. Following an initial baseline assessment (conducted in person), participants are asked to participate in 2 subsequent in-person visits every 9 months, totaling 3 visits across an 18-month study period. The participants are also asked to participate in a daily remote EMA survey occurring for 14 days after the in-person study assessment. As has been our long-standing practice for work with this heterogeneous community in Chicago, we are using an HIV status–neutral approach with Black SMM and TW to increase inclusivity and minimize the risk that participation in the study could disclose HIV status [101]. The HIV status–neutral approach (also known as an integrated continuum) is a person-centered approach that uses the same treatment approach for all individuals, regardless of HIV serostatus. This approach has been noted as a strategy that works toward achieving health equity among marginalized populations and is in line with ongoing calls for prioritizing the social determinants of health in health equity research [101,102]. Relatedly, all study procedures are identical for participants regardless of their HIV serostatus.

### Hybrid Observational-Implementation Approach

As part of the observational-implementation hybrid approach, we are preparing to conduct multiple activities as part of N2P2 that will provide data on implementation constructs related to evidence-based interventions that address salient health issues impacting Black SMM and TW. For example, we are currently collecting data that will inform how to improve the delivery of evidence-based interventions to reduce alcohol use among Black SMM and TW who could benefit from reducing their drinking. Specifically, we are developing a Discrete Choice Experiment (DCE) [103,104] to determine preferences for alcohol interventions among N2P2 participants who have an Alcohol Use Disorders Identification Test-Consumption score ≥4 [105,106]. DCE’s are a preference elicitation method that asks participants to complete multiple-choice tasks (eg, paired comparisons of profiles A vs B) with different levels and combinations of questions to compare various attributes (ie, key intervention design features). The DCE that we are currently developing will ask N2P2 participants for their preferences regarding the delivery of several evidence-based alcohol interventions, including pharmacotherapy [107,108], behavioral therapies [109,110], and mobile health interventions [111,112]. Attributes that will be included in the DCE tasks include type of intervention, person delivering the service, delivery settings, and communication platforms. We will consult with the N2P2 community advisory board as part of the formative work for refining the DCE, and it will be incorporated into the second wave of N2P2 data collection using a logic step in the survey.

In addition to the DCE on preferences for alcohol intervention delivery, we will also apply a human-centered design approach [113] to a subsample of N2P2 participants to create journey maps of Black SMM and TW experiences as they access HIV prevention and care settings. We are also using the same approach to understand the experiences of health care providers as they deliver HIV prevention and care services to Black SMM and TW. These data will then be used to inform how to tailor alcohol interventions to be more contextually informed to better suit the needs of Black SMM and TW and how to integrate them into community-based and clinical HIV prevention and care service settings. In subsequent waves of data collection, we plan to explore the collection of data on implementation constructs related to evidence-based interventions that address cannabis use, such as cognitive behavioral therapy. In addition, as recruitment settings for N2P2 seeds include HIV testing and substance use treatment facilities, and referral participants are likely to have accessed these services as well, we will also collect data from N2P2 participants about their experiences with these interventions. Collectively, these data can be used to better understand the implementation of evidence-based interventions and related determinants.

### Recruitment

**Figure 2:**
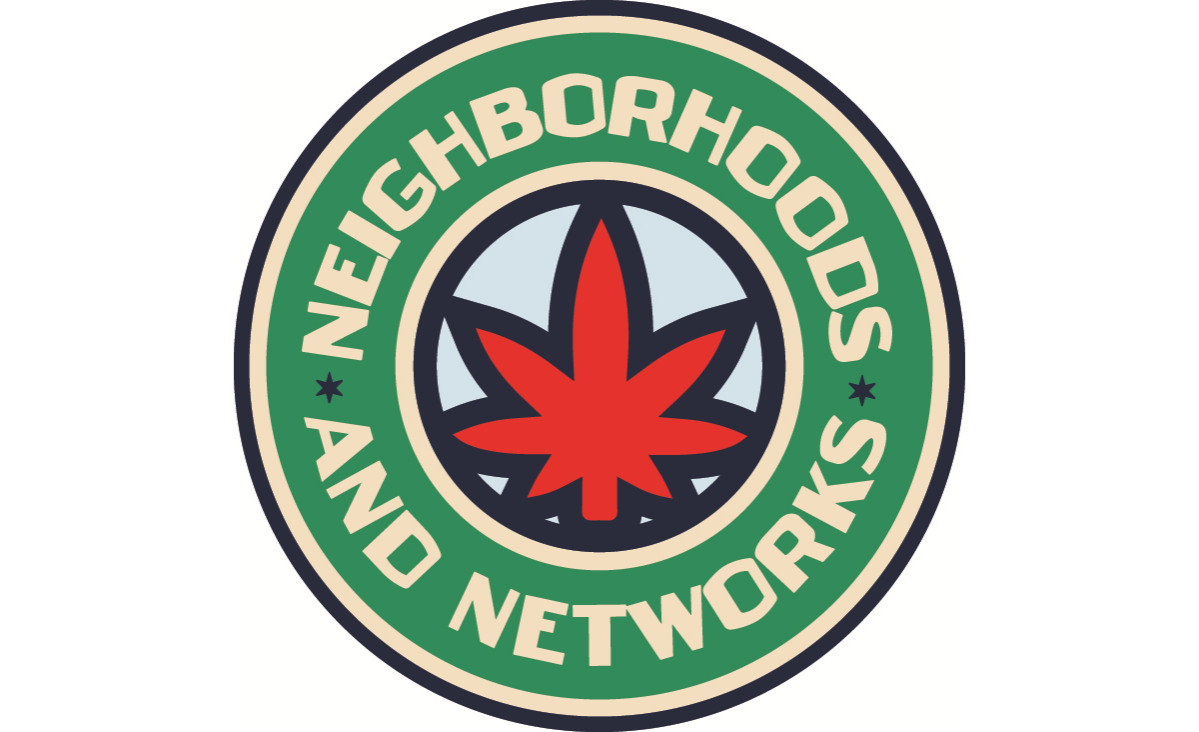
Neighborhoods and Networks Part 2 (N2P2) study logo.

Soft-launch recruitment for N2P2 commenced in September 2022. Respondent-driven sampling (RDS), a systematic form of snowball sampling that leverages existing social networks, is being used for recruitment [114]. RDS has previously been used to sample marginalized populations [115], including Black SMM and TW [49,116]. A total of 60 nonrandomly selected “seed” participants will be recruited from a variety of community sources with attention paid to diversity in geography and prior involvement in research and substance use (eg, recruiting seeds who use methamphetamine) [117,118]. In the cohort before N2, uConnect (n=612), we achieved a representative sample using approximately 60 seeds, which generated a large sample (n=620) [119]. Although a diverse sample was generated, homophily was achieved across several demographics in fewer waves (including some nonproductive seeds) given the inclusion criteria based on race, geography, and age. We hope to replicate a successful RDS in N2P2. Study facilitators will monitor the recruitment process and track referrals along recruitment threads. During recruitment, participants will receive a flyer with a unique identifier code, allowing them to be added to the study, which will also be used to indicate who referred them. For each additional individual that a participant successfully recruits to the study, the participant will receive US $30 compensation (up to a maximum of 6 additional participants) via cash or an electronic money transfer service of their choice (eg, PayPal, Cash App, and Venmo). Recruitment materials were developed to increase awareness of the study among the community, including the development of an N2P2 study logo (Figure 2).

Eligibility criteria for inclusion in the N2P2 study include (1) assigned male sex at birth, (2) identify as being Black or African American, (3) aged 18 to 34 years, (4) report of at least 1 sexual encounter with another man or transgender woman within the past year, and (5) currently residing in the Chicago MSA with no plans to move or relocate during the proposed study period. Individuals who are unable to provide informed consent are excluded from participating in the study. Individuals who screen eligible will then be scheduled for an appointment at the study site, the Village, the Chicago Center for HIV Elimination’s, off-campus community service and research space that has served Chicago’s South Side community for over a decade and that specializes in HIV prevention and care services [120].

Regarding the eligibility criteria, we made a conscious decision to be inclusive in the N2P2 study and engage Black TW as well as Black SMM. There are multiple reasons for this decision. First, the environments in Chicago in which both Black SMM and TW predominantly live include hyper-segregated neighborhoods that create similar exposures that are major drivers of HIV [121-123]. In addition, networks are often similar and overlapping with social networks including members of the house or ball community and sexual networks including many SMM. Indeed, research has shown that Black SMM and TW are members of social and sexual networks that are associated with HIV-related outcomes in nuanced ways [15-17,124-127]. In addition, many Black TW transition later than their White counterparts, and they identify at younger ages as SMM [128-130]. The reasons for this are complex, but the situation is likely not helped by most adolescent gender programs across the United States (including in Chicago), which underuse gender-affirming hormone therapy [131,132]. Finally, the decision to intentionally include both Black SMM and TW in the N2P2 study was made with community input, including from participants in the original N2 study, and with the understanding that social and sexual networks among Black SMM and TW in Chicago significantly overlap [126,133]. Importantly, community input highlighted increasing inclusivity, with particular attention needed to ensure that survey materials, procedures, and protocols are culturally tailored and gender affirming, with appropriate skip patterns and language to ensure that community members feel included and respected.

We recognize that transgender individuals describe themselves in a variety of different ways; for the purposes of our study and in congruence with established research on TW with HIV [134,135], we define TW as individuals who were assigned male sex at birth and have a feminine gender identity. We acknowledge that it is important to note that Black SMM and TW are distinct populations that experience unique social contexts and challenges related to substance use, sleep health, and HIV-related outcomes [14,136]. Indeed, studies have demonstrated that compared with Black SMM, Black TW experience substantially higher rates of financial instability, incarceration, and HIV and STIs [137,138].

### Procedures and Measures

Assessments are conducted every 9 months for 3 waves. Assessments are conducted in person in private interview rooms at an off-campus research and service space, The Village, which is located in the same building that has been engaging Black sexual and gender minority community members for >20 years. Four different forms of data are collected at each assessment: (1) a self-report survey, (2) biological specimen collection, (3) the administration of a daily remote EMA survey, and (4) extraction of EHR data. The prospective design of N2P2 enables the study team to alter and expand future surveys based on preliminary findings, including measuring relevant implementation issues, as per the hybrid observational-implementation approach [99]. After completing the survey, blood samples are drawn by trained study staff and screened for HIV antibodies to determine participant HIV serostatus. To encourage engagement with community members from all backgrounds, reduce barriers to entry, and increase accessibility, participants have the option to complete assessments remotely on Zoom (Zoom Video Communications), thus reducing the in-person requirement of participation to only collection of the biospecimens, which are scheduled at a separate date and time following survey completion. At the end of the assessment, survey technicians confirm contact information, schedule follow-up visits, provide instructions on completing the daily EMA survey, and disburse compensation for completion of the survey (US $100) and provision of biological samples (US $50) in cash or other preferred mechanism for compensation. Finally, as we did for the original N2 study [139], we continue to seek permission to access EHR data and health department data of each participant via release of information forms and subsequently link these data with the participant’s study identification number. Details on each type of data collection are described in the following sections.

### Self-Report Survey

#### Overview

The self-report survey was developed by the study investigators in tandem with the University of Chicago Survey Laboratory. Of note, certain constructs from N2 Part 1 continue to be used in this N2P2 study, allowing for robust longitudinal analyses. Trained survey technicians obtain written informed consent before participants complete a survey using computer-assisted participant interview (CAPI) technology. Self-report survey measures assessed using CAPI technology are described by construct in the paragraphs below and summarized in Table 1.

**Table 1:** Neighborhoods and Networks Part 2 measures assessed via computer-assisted participant interview.

Construct		Measurement	Reliability (Cronbach α)
Demographics (age, sex assigned at birth, height, weight, gender identity, sexual orientation, sexual attraction, history of incarceration, housing status, education, employment, income, and health insurance status)		Sociodemographic questionnaire	N/A^a^
**Potential moderating and mediating variables**			
	Neighborhood factors	Neighborhood preferences: questions adapted from the Belgian Environmental Physical Activity Study [140]	N/A
	Neighborhood factors	Neighborhood disorder and violence scales [141,142]	.70 [141] and .913-.921 [142]
	Network factors	Network composition*:* questions adapted from the Chicago Community Adult Health Study [143]	.85
	Network factors	Network size and PrEP^b^ use*:* questions adapted from the National HIV Behavioral Surveillance System [144]	N/A
	Obesity	Sociodemographic questionnaire	N/A
	Anxiety and depression	Patient Health Questionnaire-4 [145]	.85
	Self-efficacy	Sherer Self-Efficacy Scale [146]	.71-.86
	Race-based harassment	Black Men’s Experiences Scale [147]	.86
	Intersectional discrimination	Intersectional Discrimination Index [148]	.70-.72
	Interpersonal violence	Questions adapted from the original Neighborhoods and Networks cohort study	N/A
	Social support	Medical Outcomes Study Social Support Scale [149]	.91-.96
	Loneliness	Loneliness Scale for Emotional and Social Loneliness [150]	.70-.76
**Implementation data**			
	Preferences for alcohol interventions	Discrete choice experiment [103,104]	N/A
**Exposures**			
	Cannabis use	Daily Sessions, Frequency, Age of Onset, and Quantity of cannabis use inventory [151]	.69-.95
	Cannabis use	Cannabis Use Disorders Identification Test-Revised [152]	.73
	Cannabis use	Marijuana Motives Measure: Cannabis Coping Motives Subscale [153]	.80-.89
	Cannabis use	Questions adapted from The International Cannabis Policy Study [154]	N/A
	Alcohol use	Alcohol Use Disorders Identification Test [106]	.88
	Other substances	Questions adapted from the 2020 National Survey on Drug Use and Health [155]	N/A
	Other substances	Drug Abuse Screening Test [156]	.71-.94
	Sleep	*Sleep duration and quality:* Pittsburgh Sleep Quality Index [157]	.83
	Sleep	*Sleep-related problems:* Functional Outcomes of Sleep Questionnaire [158]	.87
**Primary outcomes**			
	Antiretroviral therapy adherence	Wilson self-report scale [159]	.83
	Retention in HIV care	Missed visit proportion	N/A
	PrEP uptake	PrEP uptake: questions adapted from the National HIV Behavioral Surveillance System [144]	N/A
	PrEP Adherence	PrEP adherence: Wilson self-report scale [159]	.87
**Secondary outcomes**			
	Sexual behaviors	Questions adapted from the National HIV Behavioral Surveillance System [144]	N/A

^a^N/A: not applicable.

^b^PrEP: pre-exposure prophylaxis.

#### HIV Care

Self-reported HIV care outcomes include ART adherence and linkage to HIV care, which are assessed using validated measures [144,159].

#### PrEP Cascade of Care

To assess self-reported PrEP outcomes, we are providing participants with an adapted description of PrEP used successfully with samples of SMM and TW vulnerable to HIV [160]. The PrEP cascade of care measures include *current PrEP use* [144], *PrEP modality* (pill or injection)*, PrEP initiation and discontinuation, and PrEP adherence* [159].

#### Cannabis Use and Other Substance Use

Cannabis use is being assessed via multiple validated and reliable measures that describe the frequency of use [151], modes of use [151], timing of use [151], risk of disordered use [152], motivations for cannabis use [153], and reasons for seeking cannabis treatment (if sought out by participants) [154]. The use of other substances such as alcohol, stimulants, heroin or opiates, and nicotine is also assessed and described in terms of frequency, method of consumption, and risk of disordered use [105,155,156].

#### Sleep

Sleep health outcomes include the self-reporting of sleep duration [157], sleep quality [157], and sleep-related problems (eg, apnea and trouble falling asleep or insomnia) [158].

#### Sexual Behavior

We are measuring partnered sexual behavior in the past 3 months [161], including characteristics of sexual partners (eg, gender, relationship type, HIV status, PrEP use, and undetectable viral load), sexual acts, and condom use.

#### Moderating Variables

Additional relevant individual-, network-, and neighborhood-level characteristics will be assessed as moderators of the impact of cannabis use on PrEP outcomes. Individual-level characteristics will include sociodemographics, reasons for using cannabis, cannabis use expectations, intersectional discrimination [148], and race-based harassment [147]. Obesity will also be assessed as a potential moderator for analyses involving sleep health as an exposure variable. Social and sexual network characteristics will include the racial and ethnic composition of network members, network size, network substance use, and network PrEP use. Neighborhood-level characteristics will include socioeconomic disadvantage, racial and ethnic composition, and locations of dispensaries. Many of these measures were captured in the original N2 study. Whenever feasible, preexisting N2 data will be used.

#### Covariates

Data are being collected on the following relevant sociodemographic variables: age, ethnicity, sexual orientation, sexual attraction, socioeconomic status (eg, education, income, and employment), current living situation (eg, housing instability), nativity, relationship status, mental health, history of incarceration, health insurance status, and existing or recent STIs (eg, chlamydia, gonorrhea, and syphilis).

### Biological Specimen Collection

Collected blood samples are screened on site for HIV and syphilis, after which the plasma from each sample is objectively measured for quantitative levels of cannabis metabolites via liquid chromatography–tandem mass spectrometry [74]. These metabolites will also be measured using solid-phase extraction and gas chromatography–negative ion chemical ionization mass spectrometry [162]. The detection of cannabis metabolites in plasma will be used to confirm self-reported cannabis use by the study participants. In addition, we will explore the utility of using plasma levels of tetrahydrocannabinol to stratify participants into cannabis use groups (eg, heavy user or moderate user) [163]. Cytokine and chemokine levels are also assessed in the systemic circulation (plasma samples) using the Human Inflammation Panel via the LEGENDplex assay (BioLegend, Inc), which measures 13 different cytokines or chemokines, including those relevant to HIV infection [164].

After instruction by study staff, the participants also self-administer 3 rectal swabs and 1 oral swab. The 1 oral swab and 1 of the 3 rectal swabs are tested on site for chlamydia and gonorrhea nucleic acid amplification tests [165]. The remaining 2 rectal swabs are evaluated for the levels of proinflammatory cytokine and chemokine in the rectal mucosal secretions. Finally, participants are asked to provide a urine sample, which is used to conduct on-site toxicological screening of marijuana, methamphetamine, opioids, and cocaine as well as chlamydia and gonorrhea nucleic acid amplification tests. Participants who test positive for HIV or STIs or who are interested in PrEP, primary care, or substance use treatment are linked to care with services available in the same building as the data collection. A summary of the biological measures is presented in Textbox 1.

**Biological data in Neighborhoods and Networks Part 2 data collection.**

**Constructs and measurements**

- Cannabis use
  Biological presence in blood or urine samples
- Other substances
  Biological presence in blood or urine samples
- Sexually transmitted infections
  Biological data for chlamydia, gonorrhea, or syphilis in urine, blood, saliva, or rectal mucosa samples
- Inflammation
  Biological data on cytokine and chemokine levels in systemic circulation and rectal mucosa

### EMA Survey

The EMA survey is administered over a 2-week period following the in-person assessment. If participants are unable to complete the EMA survey using their personal phone, the study team sends the EMA survey via email. Participants receive a daily notification through their cell phones at the same time each day asking to complete the brief survey. The participants have 2 hours to complete the survey. Participants receive an additional reminder notification 1 hour before the survey expires as well as personalized reminder text messages from the study staff. Daily assessments (rather than more frequent assessments) were chosen to minimize participant burden, based on feedback from community members who participated in the original N2 study. Participants receive US $2 for each daily survey completed within the first week and US $3 for each daily survey completed during the second week. The participants also receive a US $5 bonus if all 14 days are completed. The EMA survey includes multiple measures regarding policing, substance use, HIV outcomes, and mental health. Example questions and response options from the EMA survey are listed in Table 2.

**Table 2:** Ecological momentary assessment survey measures in Neighborhoods and Networks Part 2 data collection.

Measure	Example question	Example response options
Policing	“In the past 24 hours, did you encounter someone in law enforcement that:”	“(Select all that apply) Pushed or grabbed you?; Followed you?; Made you feel unsafe?; etc.”
HIV outcomes	“In the past 24 hours, did you take a daily PrEP^a^ pill”	“Yes; No”
Cannabis use	“In the past 24 hours, how many times did you use cannabis?”	“(Numerical drop-down selection) 1-7+”
Other substance use	“In the past 24 hours, did you use any of the drugs listed below?”	“Heroin (Smack, Junk, Dope); Fentanyl (China White, China Girl, Tango); Benzos; Methamphetamine (Speed, Meth, Crystal, Crystal Meth, Ice, Crank); Cocaine (Coke, Snow, Crack, Rock); Prescription opioids; Unknown combination of opioids; Other; None of the above”
Alcohol use	“In the past 24 hours, how many drinks of alcohol did you have?”	“(Numerical drop-down selection) 1-7+”
Anxiety and depression [145]	“In the past 24 hours how often were you bothered by the following problems (if at all)?”	“Not at all; Several times throughout the day; More than half the day; Nearly all day”

^a^PrEP: pre-exposure prophylaxis.

### EHR Data

#### Overview

As we did for the original N2 study [139], we continue to seek permission to access the EHR data of each participant via release of information forms from a local partnering health clinic and surveillance data from the health department and subsequently link these data with the participant’s study identification number. The outcomes described in the following subsections will be extracted from participants’ EHRs (Textbox 2).

**Electronic health record data in Neighborhoods and Networks Part 2 data collection.**

**Constructs and measurements**

- Viral suppression
  Biological viral load
- Retention to HIV care
  Missed visit proportion
- Pre-exposure prophylaxis (PrEP) outcomes
  PrEP persistence and type of PrEP product

#### Viral Suppression

Viral suppression data will be obtained from the participants’ EHRs. Viral suppression is defined as having a viral load of ≤200 HIV RNA copies/mL.

#### Retention to HIV Care

Retention to HIV care is measured by calculating a missed visit proportion, or the proportion of total scheduled visits (eg, nonacute appointments with an ART-prescribing provider) that are missed across the 27-month follow-up period [166,167]. Appointment and visit history to calculate the missed visit proportion will be collected from each participant’s EHR. To permit longitudinal analyses, we will divide the 27-month follow-up period into three 9-month sections and record, for each participant, whether they completed or missed their scheduled appointment during that section.

#### PrEP Outcomes

Several PrEP-related outcomes will be obtained from the participants’ EHR data, including PrEP persistence, which will be measured in several ways (eg, number of dedicated PrEP visits and number of prescriptions for PrEP) [168]. The type of PrEP product will also be collected, although we expect limited uptake of long-acting injectable PrEP in this community owing to a number of social and structural barriers; notably, these barriers are repeatedly observed with each new biomedical advance purportedly developed for community members classified as most vulnerable [169,170].

### Data Management

Each N2P2 participant receives a unique study identification number, which is used to link all 3 forms of the primary data into a centralized database in the same manner as our original N2 study [139]. Data will be analyzed with the aim of maintaining participant anonymity. Access to the participant identification number, which links data to unique identifiers, will be restricted to designated members of the research team. All survey, biological, EMA, and EHR data will be uploaded to the encrypted University of Chicago servers and will be backed up on a weekly basis.

### Data Triangulation

For data triangulation, objective measures will be prioritized, using previously validated decision rules for combining objective biomarker data with self-reported measures, an approach that has been used in other studies with regard to substance use [171,172] and that we will adapt as needed. This approach will allow us to account for the limitations of respective measures (eg, inaccuracy of self-report [173] and varying rates of metabolizing substance use [174]) and also allow us to identify relevant categories, as has been done in other studies [74,175]. Using multiple measures to assess constructs, particularly those regarding sensitive topics, is superior to relying on a single method [176].

### Statistical Analyses

The longitudinal design of N2P2 will allow researchers to evaluate changes in constructs over time as well as cross-sectional, series cross-sectional, and longitudinal associations between exposures and outcomes [177]. We will use generalized linear models to evaluate the associations between relevant exposures and outcomes. These models will include the appropriate time-invariant and time-varying covariates to adjust for confounding of each exposure-outcome relationship. In addition, we will apply generalized estimating equations to account for repeated measures within individuals across study visits. For analyses that assess mediation, we will use statistical tests that leverage the latest statistical methodological advances in the causal inference field and that test for and estimate the effect of mediation (ie, the natural indirect effect). Our inferences will support a causal mediation interpretation if the covariates effectively control for confounding in 4 key relationships: the impact of the candidate exposure on the outcome, the effect of the candidate exposure on the candidate mediator, the influence of the candidate mediator on the outcome, and if the candidate exposure has no impact on any confounding variables related to the effect of the candidate mediator on the outcome. We recognize that this approach is among various mediation analysis methods, and we will also consider other commonly applied approaches [178]. To assess effect modification, we will conduct regression analyses by adding 2-way interaction terms (eg, cannabis use × moderator) to the previously described generalized linear models. We also plan to evaluate cross-level interactions to determine whether our 2-way interaction analyses vary by other variables in the associations between our candidate exposures and outcomes. We further plan to use additional modeling approaches, including egocentric analysis, mesolevel sociometric analysis, multilevel (affiliation network) modeling, 2-mode person or place network modeling, synergistic place or network models, and spatial autocorrelation regression modeling. In some analyses, moderating variables that are considered confounders will be adjusted for as covariates.

### Power Calculations

We have adequate power to address our primary study aims. With a planned baseline sample size of 600 Black SMM and TW with 80% attrition over the study period and assuming α=.05 and power=0.80, we estimate that we will have 80% power to detect Cohen *d* effect sizes of 0.40 in differences of continuous HIV-related outcomes (based on distributions of those outcomes in N2 baseline data) in the highest versus lowest quintiles of exposure in the cohort. This is a conservative calculation, as it does not account for the adjustment of covariates, which can serve to increase power. To maximize the sample size and minimize potential selection bias as a result of loss of participants who do not attend all follow-up waves, we will use a pooled analysis approach and include participants who attend at least 1 of the 3 waves over the study period. A total of 600 participants will contribute 1800 potential observations across waves. This approach has been used in our prior work and in other cohort studies to efficiently maximize analytic capabilities [179]. Moderation analyses may not be optimally powered, especially to examine cross-level interactions. Mediation analyses may also not be optimally powered. However, to mitigate power issues, we plan to dichotomize sociodemographic characteristics when possible and appropriate [180]. Moreover, the potential mechanisms linking study exposures to HIV-related outcomes will be the focus of future research proposals (ie, adequate power will be prioritized).

### Governance and Organizational Study

Research strategies and protocols for the N2P2 study were developed using a community-based participatory approach [181] where study staff members regularly meet with the community advisory board to guide the study. These meetings involve receiving community feedback on survey assessments and protocols, presenting progress reports on the study, discussing preliminary data, and determining the best practices for dissemination to community members and policy makers. In addition, 1 staff member who is from the Chicago Black SMM and TW community further guides community engagement through periodic social events and social media development. Moreover, we have also created a scientific advisory board to provide expert feedback on the overall study design and methodology of N2P2, thereby supporting the collection and analyses of high-quality data.

### Ethical Considerations

This study was conducted in accordance with the guidelines of the Declaration of Helsinki. The N2P2 study protocol has been approved by the institutional review board (16-1419) of the University of Chicago (institutional review board of record), and a reliance agreement has been established with Columbia University. Written informed consent is required from all participants before participation in the study. Benefits to participation in the study include sharing the results of HIV serostatus and STI testing at multiple time points as well as referral to relevant health services when necessary (substance use treatment and mental health).

## Results

The N2P2 study was funded in August 2021 by the National Institute of Drug Abuse (R01DA054553 and R21DA053156) and National Heart, Lung, and Blood Institute (R01HL160325), and approved by the institutional review board in September 2022. Data collection was launched in November 2022. As of manuscript submission, 431 participants have been enrolled. Recruitment and enrollment for the first wave of data collection are currently ongoing.

## Discussion

### Expected Findings

In summary, the N2P2 study applies innovative methods to comprehensively explore the impact of substance use and sleep health on HIV-related outcomes among an HIV status–neutral cohort of Black SMM and TW in Chicago. This study applies an observational-implementation hybrid design to prioritize the expedited translation of findings into public health impact, a critical priority among populations such as Black SMM and TW that experience long-standing inequities with regard to HIV and other health-related outcomes owing to systemic racism, homophobia, and transphobia. N2P2 directly builds on the findings of the original N2 study among Black SMM and TW in Chicago. These findings contribute to a better understanding of how multilevel (eg, individual, network, and neighborhood) factors are associated with HIV prevention and care outcomes among Black SMM and TW [139,182,183]. Relatedly, the N2 COVID-19 study contributed additional findings related to factors associated with PrEP and ART use, among other health outcomes and health behaviors, at the initial peak of the COVID-19 pandemic [50,184-186].

A major goal of the N2P2 study is to inform the implementation of evidence-based practices that already exist to address many of the issues that we are studying. For example, we observed a high prevalence of heavy drinking in the original N2 study, which aligns with other studies among Black SMM and TW [187]. We also observed from the N2 COVID-19 study that alcohol use increased among Black SMM and TW during the pandemic [50], as it did among the general population [188,189]. However, very few individuals who could benefit from alcohol treatment receive it [190,191], despite the availability of multiple evidence-based interventions [107], indicating a clear implementation gap. Therefore, in this observational cohort study, we are working to incorporate elements of implementation science related to evidence-based interventions that address alcohol use. We have named this the *hybrid observational-implementation approach*, and we have previously described how this practical approach can be applied to observational epidemiologic research to make it more consequential by contributing more directly to the growing implementation science movement [99]. This may also help to make the research pipeline more efficient and faster [192] and help observational research achieve its intended public health impacts, an innovation that is especially important for health equity research. In future waves of the N2P2 study, we intend to expand this work to address other salient health issues, particularly those that we learn about through findings from the existing N2 study data, or in the upcoming waves of N2P2 data collection.

In addition, given the longitudinal nature of our data collection, we are able to continue examining COVID-19 as well as recently emerged STI threats (eg, mpox [193]), evolving STI epidemics such as syphilis, and other infections that disproportionately impact these communities (ie, meningococcus). This can be an important extension for future N2P2 waves, as it is known that there are clear racial and ethnic differences in new and emerging infectious disease epidemics, including among Black SMM and TW [194,195].

Maintaining meaningful community engagement at each step of the research process and enhancing those efforts are top priorities in the N2P2 study, an approach used in the original N2 study. For example, community members provided iterative feedback that has informed this study’s design. In addition, the study team organized several community dinners and social events (eg, study relaunch event at the Village) to share the findings of the original N2 study and further strengthen community trust and inclusion. We aim to continue prioritizing shared decision-making and using innovative community-based research approaches (eg, crowdsourcing and human-centered design) as the N2P2 study progresses forward [196].

Relatedly, many papers from the original N2 study were led by trainees and early-stage investigators (eg, postbaccalaureate research coordinators, master’s-level students, PhD students, and postdoctoral fellows), including those who hold minoritized identities, particularly trainees who hold the same intersectional identities as those in the N2 study. This has enhanced our ability to situate interpretations of data in the community context and obtain high-quality data. For example, because many research coordinators hold similar intersectional identities as N2 study participants, we were able to build trust with the community. This hopefully will contribute to less misclassification in self-reported measures and a greater willingness to adhere to study protocols. In N2P2, we plan to continue this work, especially because we recognize the harms that can arise from power differentials between researchers and study participants [197]. We view this component of our work as part of our intersectional and health equity–focused research program, which aims to ultimately achieve better health outcomes among Black SMM and TW [198,199].

Finally, we note that the N2P2 study currently does not have funding for recruitment in the Southeastern United States, which was a component of the original N2 study. We are actively working to acquire funding to study Black SMM and Black TW in the Southeastern sites included in N2, including New Orleans and Baton Rouge, given that the burden of the domestic HIV and AIDS epidemic is heaviest among Black SMM and TW living in that part of the country [200].

### Strengths and Limitations

The N2P2 study has some limitations. First, our sample of Black SMM and TW is sampled exclusively from the Chicago MSA and may have limited generalizability to other geographic contexts, such as smaller cities or rural areas. We decided to continue our focus on the Chicago MSA as >80% of the US population lives in urban settings [201]. Chicago shares similar contextual and environmental characteristics with many other hyper-segregated EHE jurisdictions in the United States, such as Houston and Detroit. In addition, owing to the duration of the cohort, our study’s ability to assess causality may be limited by participant loss to follow-up. This limitation is particularly significant for minoritized populations such as Black SMM and TW because of the structural barriers that these groups experience. For example, in the original N2 study cohort, 70% of the participants had an annual income of <US $25,000 and 35% reported unstable housing. These structural barriers have also been shown to be related to HIV-related outcomes [48], and they also serve as barriers to participation in research. To mitigate this challenge, participant retention is prioritized within the study’s community integration efforts as well as through protocol-based approaches such as recording participant contact information and consistent efforts to connect with participants and remind them of their engagement with the study. Moreover, owing to social desirability bias, it is possible that substance use, sexual behaviors, and PrEP and ART nonadherence are underreported. We aim to reduce the effects of this bias by obtaining objective biomarker data and using CAPI to collect potentially sensitive information. Finally, we recognize heterogeneity in Black SMM and TW and the importance of meaningful intersectional analyses; however, because we are not intentionally focusing on this heterogeneity, we may have limited power for subgroup analyses such as by ethnicity and socioeconomic status. For example, in the original N2 study, although we have variation in socioeconomic status, only 6.6% of the sample reported being Latinx or Hispanic ethnicity. We are also making efforts to ensure variability in other factors that are related to health, such as our intentional geographical sampling approach, and collect data on experiences that are relevant to Black SMM and TW, such as membership in the house or ball community and other queer family structures.

### Conclusions

These limitations are offset by many notable strengths. In N2P2, we are using a unique and innovative approach to simultaneously explore multiple salient questions about what potentially drives the long-standing HIV-related inequities experienced by Black SMM and TW [1,9]. Combining resources to conduct a comprehensive study, such as N2P2, marks a potentially more efficient use of resources, one that may reduce the logistical challenges associated with establishing independent cohorts to address each of these topics independently. The inclusive HIV status–neutral approach minimizes potential stigmatization and enhances the potential to connect participants with relevant HIV prevention and treatment services. Furthermore, the multiple innovative methods that are being used to collect primary data in N2P2, including EMA, biobehavioral overlap, preference elicitation, and human-centered design, will allow a more holistic understanding of how substance use, sleep, and HIV-related outcomes interrelate. Similarly, because of the pressing need for additional research on the HIV epidemic, specifically within Black SMM and TW communities, the study findings from N2P2 may have a substantial potential public health impact. As such, the N2P2 study provides a pivotal opportunity to better understand the impact of substance use and sleep on HIV among Black SMM and TW, which may inform the development of future evidence-based culturally relevant interventions and policy approaches.

## Appendix

Multimedia Appendix 1 Peer-reviewed reports from NIH.

## Abbreviations

ART: antiretroviral therapy
CAPI: computer-assisted participant interview
DCE: Discrete Choice Experiment
EHE: ending the HIV epidemic
EHR: electronic health record
EMA: ecological momentary assessment
MSA: metropolitan statistical area
N2: Neighborhoods and Networks
N2P2: Neighborhoods and Networks Part 2
PrEP: pre-exposure prophylaxis
RDS: respondent-driven sampling
SMM: sexual minority men
STI: sexually transmitted infection
TW: transgender women

## Data Availability

Once data are collected, they will be stored in a public repository hosted by Data Commons. In addition, investigators can obtain data via an email request to the Neighborhoods and Networks Part 2 staff.
