# Supplementary material for: HIV Prevention and Care Among Black Cisgender Sexual Minority Men and Transgender Women: Protocol for an HIV Status–Neutral Cohort Study Using an Observational-Implementation Hybrid Approach
Source: JMIR Res Protoc. 2023 Dec 1;12:e48548. doi: 10.2196/48548 (PMC10724817; doi:10.2196/48548)
Supplement: Multimedia Appendix 1 [file resprot_v12i1e48548_app1.pdf]

SUMMARY STATEMENT

(Privileged Communication)

PROGRAM CONTACT:
Richard Jenkins
301-443-6504
jenkinsri@mail.nih.gov

Release Date: **03/04/2021**
Revised Date: **03/04/2021**

Application Number: **1 R01 DA054553-01**

Principal Investigators (Listed Alphabetically):

DUNCAN, DUSTIN T (Contact)
KNOX, JUSTIN

Applicant Organization: COLUMBIA UNIVERSITY HEALTH SCIENCES

Review Group: **ZDA1 TXT-V (09)**
National Institute on Drug Abuse Special Emphasis Panel
PrEP for HIV Prevention among Substance Using Populations (R01 - Clinical Trial Optional)
AIDS - EXP. REV.

Meeting Date: **02/11/2021**
Council: **MAY 2021**
Requested Start: **07/01/2021**
RFA/PA: **DA21-024**
PCC: **CV/RJP**
Dual PCC: **A22**
Dual IC(s): **AI**

Project Title: **Cannabis use, PrEP and HIV transmission risk Among Black MSM in Chicago**

SRG Action: **Impact Score:36**
Next Steps: **Visit https://grants.nih.gov/grants/next_steps.htm**
Human Subjects: **30-Human subjects involved - Certified, no SRG concerns**
Animal Subjects: **10-No live vertebrate animals involved for competing appl.**
Gender: **3A-Only men, scientifically acceptable**
Minority: **2A-Only minorities, scientifically acceptable**
Age: **6A-Children and Adults, scientifically acceptable**

Project Year	Direct Costs Requested	Estimated Total Cost
1	498,743	816,098
2	499,473	817,292
3	499,257	816,939
4	499,541	817,404
5	499,839	817,891
TOTAL	2,496,853	4,085,624

1R01DA054553-01 Duncan, Dustin

AUTHENTICATION OF KEY BIOLOGICAL AND/OR CHEMICAL RESOURCES UNACCEPTABLE COMMITTEE BUDGET RECOMMENDATIONS

RESUME AND SUMMARY OF DISCUSSION: This new application entitled “Cannabis use, PrEP and HIV transmission risk Among Black MSM in Chicago” is submitted in response to RFA-DA-21-024: PrEP for HIV Prevention among Substance Using Populations (R01 - Clinical Trial Optional) by Columbia University Health Sciences with Drs. Dustin Duncan and Justin Knox as the Principal Investigators (PIs). This multiple PI application proposes to assess cross-sectional and longitudinal associations between cannabis use on PrEP outcomes and HIV transmission risk in Black MSM in Chicago, Illinois.

During the discussion, the panel notes some strengths. The reviewers agree that the application is significant because it focuses on the key vulnerable population of Black MSM to study their PrEP outcomes in relation to high prevalence of cannabis use. Furthermore, it is innovative to build the study on an on-going cohort in a priority jurisdiction of Ending the HIV Epidemic initiative. Linking different biobehavioral measures (e.g., cannabis use by metabolites in plasma, PrEP adherence by dried blood spot, and sexually transmitted infections by blood and rectal swabs) with Ecological Momentary Assessment (EMA) is also innovative. The investigative team has a history of collaboration with expertise in infectious disease, HIV prevention, substance use, sexual minority population and epidemiology. The research team has a track record of strong recruitment with this study population. The environment is excellent and suitable to carry out the proposed research.

The application also has some weaknesses. The preliminary data for an association between cannabis and HIV acquisition are conflicting and the hypothesis is not well articulated. The panel mentions that it may be a missed opportunity to focus on a single site in Chicago instead of including more southern sites from the parent study especially given the rising HIV incidence in the southern United States. Several concerns are raised about the approach. The application does not adequately explain how data analyses would be conducted to incorporate simultaneous use of other substances with cannabis. Furthermore, it is not clear how the results of the different biobehavioral measures will be combined in the analyses. The statistical plan is underdeveloped and as such the investigative team could benefit from the addition of a statistician with expertise in sophisticated model building and model adequacy assessment. Design of the study visits incorporating the EMA lacks details and needs further clarification. Eligibility criteria are not clear. Justification for the timeline to exceed 4 years is not adequately provided.

Based upon the evaluation of scientific and technical merit, this application received an Overall Impact/Priority score of 36.

DESCRIPTION (provided by applicant): Black gay, bisexual and other men who have sex with men (MSM) face a disproportionate burden of HIV. Focused, high-coverage PrEP in populations heavily impacted by HIV, such as Black MSM, could rapidly reduce new HIV acquisition rates; however, its uptake among at-risk populations, especially Black MSM, has been limited. Therefore, we propose to conduct urgently needed research on PrEP in a cohort of Black MSM, including on the impact of relevant behaviors, particularly cannabis use, which is highly prevalent in Black MSM. Research on the impact of cannabis use on PrEP has achieved conflicting results, and it has not been rigorously studied in Black MSM. Therefore, the proposed R01 study will assess cross-sectional and longitudinal associations between cannabis use and PrEP outcomes (e.g., use, adherence) and HIV transmission risk (e.g. biological inflammation, sexual risk behavior) using event-level and objective biomarker data

among HIV-negative Black SMM. To address these specific aims, we will conduct the Networks and Neighborhoods (N2) Cannabis PrEP Study in Chicago, IL. We will follow 250 HIV-negative participants from the original N2 cohort for an additional one-year period with 3 study waves. We will use innovative and rigorous methods, to collect additional data, such as Ecological Momentary Assessment methods and objective measures of cannabis use, PrEP use, and immune function over 14-day periods at each wave. Potential findings can impact intervention development and implementation, as well as inform policy to increase PrEP uptake and adherence, address substance use, and decrease HIV transmission rates, and disparities.

PUBLIC HEALTH RELEVANCE: This project seeks to investigate relationships between multiple aspects of cannabis use in relation to HIV prevention behaviors (e.g., pre-exposure prophylaxis [PrEP] use, adherence, and discontinuation) and (biological vulnerability to HIV infection) among Black gay, bisexual and other men who have sex with men (MSM), a population in the United States heavily impacted by HIV/AIDS.

CRITIQUE 1

Significance:	3
Investigator(s):	2
Innovation:	3
Approach:	6
Environment:	2

Overall Impact: This submission entitled “Cannabis use, PrEP, and HIV transmission risk Among Black MSM in Chicago” proposes to examine the role of cannabis use on PrEP uptake and adherence in Black MSM participating in a single site (Chicago) of an ongoing longitudinal cohort study. The MPI research team (Duncan and Knox) are both located at Columbia and propose to extend the existing multisite (Chicago, New Orleans, Baton Rouge, Jackson) Networks and neighborhoods (N2) cohort study, by following participants in Chicago for one additional year to further assess the association between cannabis use, PrEP and biologic vulnerability to HIV among black sexual minority men. The research team is strong, and this team proposes to conduct the study in a population with disproportionate risk for HIV and with high rates of cannabis use. However, the preliminary data for an association between cannabis and HIV acquisition are conflicting (e.g. some studies report a decrease in sustained PrEP use associated with cannabis; others do not apparently show an association between PrEP use and cannabis; cannabis may decrease mucosal inflammation whereas other substances increase biologic vulnerability to HIV) and the application does not clearly present an over-arching hypothesis. Overall, the proposal provides extensive background for the proposed project but not enough information about the approach. Specifically, details are unclear about the incorporation of the EMA, including timing of study visits; limited information is provided about how polysubstance use will be incorporated into analyses; sample size calculations appear to go beyond numbers in this existing cohort; the use of only a single site while three other sites (in heavily impacted areas in the South) in the parent study are not apparently included and definitions of the primary exposure and outcomes measures are not adequately specific.

1. Significance

Strengths

- Population at risk: Black MSM are at disproportionate risk of acquiring HIV.
- Chicago is an *Ending the HIV Epidemic* priority jurisdiction.

- Only 1 of 3 HIV-negative Black MSM in Chicago are using PrEP and 2 out of 3 discontinued PrEP at some time after initiating it.
- Common exposure: Among HIV negative men in the existing cohort, 2 out of 3 reported cannabis use in the past month, half of whom reported daily use.
- Increasing exposure: legalization of cannabis in multiple states across the US, including IL.

Weaknesses

- Data around associations between cannabis use and PrEP are mixed, arguing that a strong association between these two is less likely (Minor).
- Given the frequent overlap of cannabis use with other substances (e.g. stimulants, club drugs) which do have known associations with PrEP uptake and adherence, identifying an independent association between cannabis and PrEP is unlikely. Other substances only minimally addressed in background (Major).
- Cannabis use disorder is reported to be 'formerly rare' and now occurs in 20-30% of users. This is not clearly addressed. Is this due to increased availability of cannabis? New definition (or new application of definition) of CUD? (Minor)
- The assertion that the longitudinal design using biomarker, self-report and event-level data will allow for causal inference seems misplaced in an observational study.

2. Investigator(s)

Strengths

- Dr. Duncan is an expert in research involving sexual minority men, HIV, substance use; social and spatial epidemiology
- Dr. Knox has experience in infectious diseases and substance use, and has a working relationship with Dr. Duncan, having been mentored by Dr. Duncan during a recently completed a T32 fellowship. Dr. Knox was awarded a K01 in 9/2020, which may indicate it is quite fast to be MPI on an R01, but has 20 first author publications.
- Co-investigators Martins, Schneider and consultants Carrico, Rendina, and Safren are well suited to the proposal and have a history of collaboration with the MPIs and expertise in the field.

Weaknesses

- Dr. Manuzak recently joined the faculty at Tulane and apparently has not previously collaborated with the MPIs (minor).

3. Innovation

Strengths

- Combination of biologic measures (serum metabolites of cannabis, dried blood spot for PrEP) with self-reported substance use and adherence.
- Use of EMA.
- Cytokine quantification assays in plasma and rectal mucosal environment.

Weaknesses

- Unclear how the results of these different measures will be combined in analyses.

4. Approach

Strengths

- Research team has a track record of strong recruitment and retention of black sexual minority men.
- Preliminary data demonstrate experience working with population, epidemiology, immunologic studies and EMA.

Weaknesses

- Polysubstance use mentioned multiple times in the background but not explained specifically how data analyses will be conducted to incorporate simultaneous use of substances with cannabis (Major).
- Limited details are provided for some of the measures. For example, time varying covariates such as housing and incarceration, are they collected multiple times or just at baseline. Is neuropsychological testing only collected at 6 months? Is it compared to a baseline value? Rectal swabs- who collects this? Clinician? Self-collected? (Minor)
- Design of study visits is confusing. Three main study visits are described, but also incorporate 2 weeks EMA at beginning and end, then another visit after EMA. Therefore, are there actually 5 study visits? (Minor)
- Sample size calculations are concerning, since recruitment is from an existing study with N=227 HIV-negative Black MSM, but sample size needed is N=250. Will new participants be allowed to enter the study? Also, how will the estimated 42% who are already on PrEP be evaluated? (Major)
- Focus on a single site (rather than including 1 or more sites in the South from the parent study), especially given rising HIV incidence in the southern US. (Major)
- Data analyses: Need more specifics on primary exposure. How specifically will cannabis plasma levels be triangulated with EMA results? Analyses for Aim 1—unclear how many models will be tested, raising concerns for multiple testing. Are there variables that will be included *a priori*? Aim 3 analysis plan only mentions rectal cytokines but plasma cytokines mentioned earlier in proposal. (Minor)
- Primary outcome of PrEP uptake, how is this defined? Visit with PrEP provider? Receipt of PrEP prescription? (Minor)

5. Environment

Strengths

- Strong academic environments for MPIs, co-investigators and collaborators.

Weaknesses

- MPIs are not located at the single study site.

CRITIQUE 2

Significance:	3
Investigator(s):	2
Innovation:	3
Approach:	3
Environment:	2

Overall Impact: This application proposes an “add-on” study to investigate the role of cannabis use in HIV prevention among Black MSM (BMSM). The scientific premise of the proposed study is based on these aspects: 1) PrEP is key to reducing HIV epidemic among BMSM; 2) the use of cannabis is highly prevalent among Black MSM and may compromise their uptake and adherence of PrEP in HIV prevention; and 3) the specific role of cannabis in relation to the PrEP remains unclear. The overall public health impact of this study thus lies in that, if successfully implemented, it shall provide a better understanding into the potential negative effect of the cannabis use on the PrEP’s uptake and adherence among BMSM, and may help develop more effective prevention intervention program and strategies targeting this key vulnerable population.

1. Significance

Strengths

- Focusing on the key vulnerable population of BMSM to study their PrEP uptake and adherence in relation to high prevalence of cannabis use is significant.
- Building the study cohort for the ease of study recruitment and follow-up on an ongoing BMSM research project is significant.

Weaknesses

- Since young BMSM are facing an increasing trend of HIV incidences, some warrant of their representation among the study participants is needed for the research findings to yield a sufficient generalizability.

2. Investigator(s)

Strengths

- The PI, Dr. Duncun, is an experienced investigator with a well-established track record in HIV/AIDS prevention research among BMSM. He has assembled an impressive investigative team for the proposed research and is well qualified to lead the team.

Weaknesses

- A senior faculty-level statistician with experience and dedicated effort in sophisticated model building and model adequacy assessment shall strengthen the team’s ability in dealing with challenges in complex data modeling and analysis, such as handling missing data, misclassification, and measurement errors.

3. Innovation

Strengths

- Investigating the role of cannabis among BMSM in their PrEP uptake and adherence in HIV prevention is innovative.
- Building on an on-going cohort in an EHE priority jurisdiction is also innovative.

Weaknesses

- No major weaknesses identified.

4. Approach

Strengths

- Using a cohort design to investigate the role of cannabis in relation to the PrEP uptake and adherence among BMSM appears reasonable and justified.
- Building on an ongoing research to assemble the cohort appears feasible.
- Sample size and power calculation are performed on both the PrEP take and adherence. Analysis plan appears comprehensive.

Weaknesses

- To ensure a sufficient representation of young BMSM in the assembled cohort, an age-stratified enrollment plan may be needed.
- Missing information, misclassification and measurement errors need to be taken into consideration in the analysis plan.
- Loss-to-follow-up can be a challenging issue for the assembled cohort. Analysis of PrEP uptake and adherence against the potential “informative censoring” in GLM/GEE modeling should be considered.

5. Environment

Strengths

- Columbia U, U Chicago and Tulane are excellent for the proposed research.

Weaknesses

- No major weaknesses identified.

CRITIQUE 3

Significance:	2
Investigator(s):	2
Innovation:	1
Approach:	4
Environment:	1

Overall Impact: The goal of this application is to examine the impact of cannabis use and PrEP uptake and adherence in a cohort of Black MSM 16 to 34. Participants will be recruited from those enrolled in the N2 cohort in Chicago, thus is responsive to the RFP. The study is innovative and is one of the few studies to focus on the longitudinal and cross-sectional associations between PrEP and cannabis use

which is highly prevalent among Black MSM. They will also examine HIV transmission risk (biological inflammation) using event-level and objective biomarker data. The proposed study has many strengths including an excellent investigative team, strong premise, rigorous procedures and use of innovative biobehavioral measures. Despite the many strengths, the main critique that reduced enthusiasm somewhat centers on questions related to the number of participants who uptake PrEP. This is concerning given that only 18% of the participants in the parent study took PrEP and the application does not seem to acknowledge this as a potential limitation. The application provides estimates of PrEP uptake, but did not adequately support these estimates with citations or unpublished data.

1. Significance

Strengths

- A significant aspect of the study is its focus on Black MSM in Chicago, an EHE designated area.
- The study addresses existing gaps in the literature related to the impact of cannabis use, which is highly prevalent among Black MSM, and PrEP outcomes. The biobehavioral and event level data generated by the study will help to address the conflicting findings in the literature and help to address how cannabis contributes to HIV transmission in this population.
- The study leverages an existing cohort, the N2 cohort study, by extending participation of a select group of participants for an additional 12 months and will incorporate EMA over a 14-day period, objective measures of cannabis use, PrEP use, and immune function at three time periods. Adding these measures to the existing social and neighborhood data and information on social networks will provide a richer understanding of the associations among cannabis use and PrEP outcomes. This approach is responsive to the requirements of the RFP.
- The scientific premise for the work proposed in the application is strong. The application compellingly argues the importance of PrEP uptake for reducing new HIV infections in the population and the insufficient data regarding the association between PrEP and cannabis use, which is prevalent in the population. Understanding these associations is also importance given that more and more jurisdictions are legalizing cannabis use which is likely to increase consumption.

Weaknesses

- Questions regarding the number of participants who will be on PrEP during the course of the study reduces significance.

2. Investigator(s)

Strengths

- The research team is very strong and has the requisite expertise to conduct the proposed aims.
- Drs Duncan and Knox, the MPI, have a strong history of collaboration as do Drs Duncan and Schneider.
- Outstanding group of consultants whose experience’s will enrich the work proposed

Weaknesses

- Dr. Duncan’s bio could have been updated. It still has a paper from 2019 listed as in press and another with Chen as the primary author is listed as in press with no year

- Justification for Dr. Knox serving as MPI was not compelling.

3. Innovation

Strengths

- The study focuses on young Black MSM drawn from an existing cohort.
- The study uses biobehavioral measures and ties these data with EMA. For instance, cannabis use will be measured via metabolites in plasma, adherence to PrEP will be measured with dried blood spots and STI will be measured via blood and rectal swabs.
- The proposed study explores the effects of PrEP, cannabis use and sexual behavior of rectal cytokine levels that may provide some information regarding biological vulnerability to HIV infection.

Weaknesses

- None noted.

4. Approach.

Strengths

- Strong conceptual model.
- Scientifically rigorous procedures.
- They focus exclusively on Black MSM and understudied at risk population. This decision is justifiable.
- Reliable and valid biological measures that are well described and appropriate.
- Appropriate MPI plan.
- Use of EMA.

Weaknesses

- The application states that the primary outcome is PrEP uptake and adherence. However, this is a cohort study and there was no apparent description regarding how PrEP uptake would happen. Is it just going to monitor if participants start PrEP at each assessment point? What if an insufficient number of participants initiate PrEP? Its power analysis estimates an increase of 6% per year, but it did not provide adequate supporting evidence for this estimate.
- There was not enough clarity in the eligibility criteria. It is clear that they want to recruit Black MSM who are negative, but what is less clear is whether or not the participants would have to be on PrEP. This is particularly problematic given that the aims of the study are to understand the association between cannabis use and PrEP uptake and adherence. The application did not adequately address this in the section under possible limitations and responses.
- The application did not fully justify a 5-year timeline. For instance, it proposes 12 months for start-up, which may not be needed given the proposed activities and the expertise of the team. Year 5 is dedicated to data analysis and writing other research grants. This study itself can probably be done in 4 years.
- Dr. Carrico’s letter of support is not signed.

5. Environment

Strengths

- The research environment at Columbia University is outstanding.
- The University of Chicago provides an appropriate research environment because of its strong history of collaboration between the social and biological science which is important to the work proposed.
- The research environment at Tulane is also appropriate.

Weaknesses

- None noted.

THE FOLLOWING SECTIONS WERE PREPARED BY THE SCIENTIFIC REVIEW OFFICER TO SUMMARIZE THE OUTCOME OF DISCUSSIONS OF THE REVIEW COMMITTEE, OR REVIEWERS' WRITTEN CRITIQUES, ON THE FOLLOWING ISSUES:

STUDY TIMELINE: NOT APPLICABLE

PROTECTION OF HUMAN SUBJECTS: ACCEPTABLE

DATA AND SAFETY MONITORING PLAN: NOT APPLICABLE

INCLUSION OF WOMEN PLAN: ACCEPTABLE

INCLUSION OF MINORITIES PLAN: ACCEPTABLE

INCLUSION OF INDIVIDUALS ACROSS THE LIFESPAN: ACCEPTABLE

VERTEBRATE ANIMALS: NOT APPLICABLE

BIOHAZARD COMMENT: NOT APPLICABLE

FOREIGN INSTITUTION: NOT APPLICABLE

SELECT AGENTS: NOT APPLICABLE

DATA SHARING PLAN: ACCEPTABLE

MODEL ORGANISM SHARING PLAN: NOT APPLICABLE

GENOMIC DATA SHARING PLAN: NOT APPLICABLE

AUTHENTICATION OF KEY BIOLOGICAL AND/OR CHEMICAL RESOURCES: UNACCEPTABLE

- The application includes measurement of cytokines and as such should include biological and chemical resources section.

COMMITTEE BUDGET RECOMMENDATIONS:

Recommended budget modifications or possible overlap identified:

- Justification for a budget period over four years is not adequately provided.

Footnotes for 1 R01 DA054553-01; PI Name: Duncan, Dustin T

NIH has modified its policy regarding the receipt of resubmissions (amended applications).See Guide Notice NOT-OD-18-197 at https://grants.nih.gov/grants/guide/notice-files/NOT-OD-18-197.html. The impact/priority score is calculated after discussion of an application by averaging the overall scores (1-9) given by all voting reviewers on the committee and multiplying by 10. The criterion scores are submitted prior to the meeting by the individual reviewers assigned to an application, and are not discussed specifically at the review meeting or calculated into the overall impact score. Some applications also receive a percentile ranking. For details on the review process, see http://grants.nih.gov/grants/peer_review_process.htm#scoring.

MEETING ROSTER

The roster for this review meeting is displayed as an aggregated roster that includes reviewers from multiple DA Special Emphasis Panels Meetings
for the 2021/05 council round.
This roster for DA is available at:
http://public.era.nih.gov/pubroster/Reports?DOCTYPE=SEP&DESFORMAT=PDF&AGENDA_SEQ_NUM_P=411783

SUMMARY STATEMENT
(Privileged Communication)

PROGRAM CONTACT:
PETER HARTSOCK
(301) 402-1964
ph45z@nih.gov

Release Date: **04/02/2021**
Revised Date:

Application Number: **1 R21 DA053156-01A1**
Formerly: **1R21DA053156-01**

Principal Investigator

KNOX, JUSTIN

Applicant Organization: NEW YORK STATE PSYCHIATRIC INSTITUTE

Review Group: **PPAH**
Population and Public Health Approaches to HIV/AIDS Study Section
AIDS - EXP. REV.

Meeting Date: **03/11/2021**
Council: **MAY 2021**
Requested Start: **07/01/2021**
RFA/PA: **PA20-195**
PCC: **EB/PIH**
Dual IC(s): **AI**

Project Title: **Social environmental drivers of stimulant use and its impact on HIV prevention and treatment in Black men who have sex with men**
SRG Action: **Impact Score:20 Percentile:1 +**
Next Steps: **Visit https://grants.nih.gov/grants/next_steps.htm**
Human Subjects: 30-Human subjects involved - Certified, no SRG concerns
Animal Subjects: 10-No live vertebrate animals involved for competing appl.
Gender: 3A-Only men, scientifically acceptable
Minority: 2A-Only minorities, scientifically acceptable
Age: 7A-Only Adults, scientifically acceptable

Project Year	Direct Costs Requested	Estimated Total Cost
1	150,000	257,466
2	125,000	214,555
TOTAL	275,000	472,021

ADMINISTRATIVE BUDGET NOTE: The budget shown is the requested budget and has not been adjusted to reflect any recommendations made by reviewers. If an award is planned, the costs will be calculated by Institute grants management staff based on the recommendations outlined below in the COMMITTEE BUDGET RECOMMENDATIONS section.

1R21DA053156-01A1 Knox, Justin

RESUME AND SUMMARY OF DISCUSSION: The applicant proposes to examine stimulant use and other drug use among Black MSM in order to characterize stimulant use and other drug use, identify their social-environmental drivers, and identify these factors' contribution to HIV transmission. This study leverages the resources of an existing cohort, namely, the Networks and Neighborhoods Cohort Study. Reviewers were very enthusiastic about this resubmission which was very responsive to prior critiques. The premise for the application is strong; Black MSM represent one of the groups at highest risk for HIV infection; there has been an increasing use of stimulants in this population, and stimulant use has been found to interfere with prevention and care. While stimulants and other drug use, are thought to contribute to the high rate of HIV, estimates of the extent of use and kinds of drugs are lacking. A better understanding of these dynamics is essential to inform the development of effective prevention interventions; thus, underscoring the significance of this study. Reviewers lauded the many strengths of the application; including the team of investigators which was referred to as "superb;" they have the complementary expertise and experience working with this population to carry-out this research; the proposed study will add in-depth interviews around stimulant use among the cohort members to the existing data on neighborhood level characteristics as well as egocentric network data collected in the previous study. This, in addition to the plan to examine behavioral and biological mechanisms for HIV, makes for a very innovative and comprehensive approach. This is a well-crafted application; and tough a descriptive study, the design, methodology and analysis plans are very robust. Some very minor concerns were raised that did not impact the very high enthusiasm of reviewers for the application; as such, the potential impact of the application was assessed as very high.

DESCRIPTION (provided by applicant): Significance. Use of stimulants is a growing problem in the US. This growing public health crisis requires expanded research to explore its reach, drivers and impact, including on marginalized groups, such as Black gay, bisexual and other men who have sex with men (MSM), a critical population that is disproportionately impacted by HIV. Estimates of the incidence and persistence of stimulant use in Black MSM is needed, as well as research on how it co-occurs with other drug use (i.e. polysubstance use), its social-environmental drivers, and its impact on HIV transmission. Research Plan. In Aim 1, we will characterize stimulant use in an established cohort of Black MSM, including co-occurring use with other drugs (i.e. polysubstance use) and use over time (e.g., incidence, persistence). In Aim 2, we will identify network-level (e.g. disassortative racial mixing, network turnover) and neighborhood-level (e.g., social cohesion, time spent in gay neighborhoods) drivers of stimulant use in Black MSM. In Aim 3, we will assess how stimulant use impacts HIV transmission in Black MSM through HIV prevention (e.g. PrEP adherence, condom use), HIV treatment (e.g. ART adherence, viral suppression) and biological vulnerability (e.g. rectal cytokines). The ongoing Neighborhoods and Networks (N2) Cohort Study (R01MH112406; PIs: Duncan & Schneider) provides an ideal opportunity to conduct the proposed study. N2 includes 186 HIV-positive and 227 HIV-negative Black MSM living in Chicago. Data being collected include stimulant use at multiple cycles, in-depth assessments of neighborhoods (including real-time geospatial methods to track mobility within and between neighborhoods), multiple social network typologies, and HIV-related prevention and treatment behaviors. The proposed study will use N2 data to conduct Aims 1-3. We also propose to use existing N2 infrastructure to recruit 30 current stimulant-using and 10 non-stimulant-using HIV-negative Black MSM from the N2 study, and conduct in-depth interviews with them using a timeline follow-back survey focused on stimulant use and sexual risk behavior, as well as collect rectal swabs, urine and blood samples as objective biomarkers, in order to explore in-depth how stimulant use contributes to HIV transmission. The results of this study will inform the development of an R34 proposal to develop and test an intervention that addresses stimulant use and HIV in a critical population. Team. Investigators with expertise in stimulant use, HIV, social network analysis, spatial epidemiology, immunology, integration of biological and behavioral research, and mixed methods research will conduct this

research together. Public Health Impact. The proposed study will be a large, rigorous and innovative study of stimulant use, its social-environmental drivers and its impact on HIV transmission in Black MSM, a group with a heavy burden of stimulant use and HIV. The proposed study is aligned with multiple NIDA funding priorities, including NOT-DA-19-066 Epidemiology of Drug Abuse, has a high likelihood of success by leveraging an existing cohort, and will directly inform an R34 proposal to develop and test an intervention that addresses stimulant use and HIV in a critical population.

PUBLIC HEALTH RELEVANCE: Use of stimulants is a growing problem in the US, including in marginalized groups, such as in Black gay, bisexual and other men who have sex with men (MSM). The proposed scientifically rigorous, exploratory R21 study aims to increase our understanding of stimulant use and other drug use in Black MSM by using the data and infrastructure of an established cohort (the Networks and Neighborhoods Cohort Study) to characterize stimulant use and other drug use, identify it's social-environmental drivers, and assess its contribution to HIV transmission. The findings will directly inform an R34 proposal to develop and test an intervention that addresses stimulant use and HIV in a critical population.

CRITIQUE 1

Significance: 2
Investigator(s): 1
Innovation: 3
Approach: 2
Environment: 1

Overall Impact: The proposed study study is designed to characterize stimulant and polysubstance use among a predominant young (80% =<29) Black MSM (BMSM) in Chicago, identify network level and neighborhood level determinants of stimulant use, and to understand how stimulant use impacts HIV prevention, treatment and biological vulnerability (rectal inflammation). The study responds to the urgent need for understand increasing use of stimulant among young Black MSM, and its impact on HIV transmission. This resubmission adequately addressed prior critiques. One strength is to leverages the infrastructure and data from an on-gong R01 Cohort Study (N2), which was designed to understand neighborhood-level and network-level determinants of PrEP. Neighborhood, network and stimulant use measures can be readily analyzed for the purpose of the proposed study. Additional primary qualitative interview data from 30 stimulant users and 10 non-users will be collected and analyzed to achieve proposed aims. The study is guided by a well-developed conceptual framework that integrate different components of the study. Data analysis plan is adequate. Findings from the study will be presented and discussed at an Intervention Translation workshop, in preparation for future intervention development study. The investigator team is very strong. Research environment at the two performance sites are excellent.

1. Significance:

Strengths

- This study addresses HIV risk among Black MSM, a high priority group in the US
- Extant literature suggests that stimulant use in this population interferes with behavioral prevention and care continuum outcomes.

- Stimulant use's impact on PrEP uptake and adherence, especially among Black MSM, is very limited

Weaknesses

- None noted

2. Investigator(s):

Strengths

- PI Knox is a very promising early stage investigator who has a strong publication record and related training and experience. He has gained grant management skills and experience from a dissertation and K01 award, and has had a coordinator role in a related R01 study.
- PI will be supported by experienced Co-Is. Drs. Duncan, Schneider, and Hasin.
- Each investigator provides strong and complementary skills, and they have a history of collaboration.
- The team also has a consultant in immunology and three scientific advisors

Weaknesses

- None noted

3. Innovation:

Strengths

- The cell-to-society approach is not only innovative but also has immediate application value in clinical practices.
- Real time geospatial tracking and mobility assessment within and between neighborhoods

Weaknesses

- Neighborhood and network influence on substance use in general, is not new, although research of such is still needed to understand stimulant use among young Black MSM

4. Approach:

Strengths

- Leverages infrastructure and data from an existing cohort of Black MSM in Chicago. Secondary data with many needed measures about stimulant and other drug use, network-level and neighborhood-level measures will be readily available for analysis.
- Assess stimulant use impact on HIV risk through lenses of prevention, treatment and biological vulnerability may generate valuable new knowledge
- This revision addressed previous critiques about better provision to future intervention development study. Figure 1 conceptual model integrates various component of the study and illustrates potential intervention points and mechanisms for intervention. CAB members' involvement in Intervention Translation Workshop is likely to facilitate translation of study findings into intervention strategies and inform the next R34 study.
- Data analysis plan is adequate
- Mixed methods approach integrating quantitative and qualitative methods is a strength.

- Previous critique about network decay issue is well addressed

Weaknesses

- None noted

5. Environment:

Strengths

- Excellent institutional resources and research support at Columbia University and the University of Chicago

Weaknesses

- None noted

Study Timeline:

Strengths

- Not Applicable (No Clinical Trials)

Weaknesses

- Not Applicable (No Clinical Trials)

Protections for Human Subjects:

Acceptable Risks and/or Adequate Protections

Data and Safety Monitoring Plan (Applicable for Clinical Trials Only):

Not Applicable (No Clinical Trials)

Inclusion Plans:

- Sex/Gender: Distribution justified scientifically
- Race/Ethnicity: Distribution justified scientifically
- For NIH-Defined Phase III trials, Plans for valid design and analysis: Scientifically unacceptable
- Inclusion/Exclusion Based on Age: Distribution justified scientifically

Vertebrate Animals:

Not Applicable (No Vertebrate Animals)

Biohazards:

Not Applicable (No Biohazards)

Resubmission:

Adequately addressed prior critiques regarding the largely descriptive nature of the application, the connection to future intervention, and the network and neighborhood measures

Applications from Foreign Organizations:
Not Applicable (No Foreign Organizations)

Select Agents:
Not Applicable (No Select Agents)

Resource Sharing Plans:
Not Applicable (No Relevant Resources)

Authentication of Key Biological and/or Chemical Resources:
Not Applicable (No Relevant Resources)

Budget and Period of Support:
Recommend as Requested:

CRITIQUE 2

Significance: 3
Investigator(s): 1
Innovation: 2
Approach: 3
Environment: 1

Overall Impact: This resubmission application addresses an important population, problem, and intervention need; the revised application more clearly addresses how this work will be used to inform an R34 application. The revised application is mostly responsive to critiques from the first review. While the study remains largely descriptive, the addition of a more detailed explanation of how the study findings will be used to develop potential interventions for an R34 application largely mitigates this concern.

1. Significance:

Strengths

- The study addresses an important population, problem, and intervention need; the revised application more clearly addresses how this work will be used to inform an R34 application.

Weaknesses

- The revised application remains largely descriptive.

2. Investigator(s):

Strengths

- The PI and collaborating investigators are outstanding, and have the multidisciplinary skills required to successfully carry out the proposed research.

Weaknesses

- None noted by reviewer

3. Innovation:

Strengths

- The cells-to-society approach and the methods for assessing neighborhoods and networks are innovative.

Weaknesses

- None noted by reviewer

4. Approach:

Strengths

- Approaches for addressing network decay in the N2 study are detailed in the revised application and are a strength.
- The clarification of plans to use explanatory sequential design provides more cohesion between quantitative and qualitative data in the revised proposal.
- Additional attention to translation of study findings into interventions is a strength.

Weaknesses

- None noted by reviewer

5. Environment:

Strengths

- Facilities and resources at the primary and collaborating institution are well-suited to carrying out this research.

Weaknesses

- None noted by reviewer

Study Timeline:

Strengths

- Not Applicable (No Clinical Trials)

Weaknesses

- Not Applicable (No Clinical Trials)

Protections for Human Subjects:

Acceptable Risks and/or Adequate Protections

- Acceptable risks with adequate protections

Data and Safety Monitoring Plan (Applicable for Clinical Trials Only):

Not Applicable (No Clinical Trials)

Inclusion Plans:

- Sex/Gender: Distribution justified scientifically
- Race/Ethnicity: Distribution justified scientifically
- For NIH-Defined Phase III trials, Plans for valid design and analysis: Not applicable
- Inclusion/Exclusion Based on Age: Distribution justified scientifically
- Population characteristics justified based on scientific question

Vertebrate Animals:
Not Applicable (No Vertebrate Animals)

Biohazards:
Not Applicable (No Biohazards)

Resubmission:
The revised application is mostly responsive to critiques from the first review. While the study remains largely descriptive, the addition of a more detailed explanation of how the study findings will be used to develop potential interventions for an R34 application largely mitigates this concern.

Applications from Foreign Organizations:
Not Applicable (No Foreign Organizations)

Select Agents:
Not Applicable (No Select Agents)

Resource Sharing Plans:
Not Applicable (No Relevant Resources)

Authentication of Key Biological and/or Chemical Resources:
Not Applicable (No Relevant Resources)

Budget and Period of Support:
Recommend as Requested:

CRITIQUE 3

Significance: 1
Investigator(s): 1
Innovation: 1
Approach: 3

Environment: 1

Overall Impact: A methodologically cutting-edge study of the relationship between stimulant use and HIV transmission, in the context of multiple levels of risk factors and social and sexual network characteristics. The overall impact of this proposal has been substantially enhanced in this resubmission through a strengthened focus on utilizing project results for intervention and policy design. In particular, specific aim 2 is likely to generate ground-breaking insights into HIV transmission among Black MSM: contributions of neighborhood and network characteristics to transmission risk. This R21 leverages an ongoing NIH-funded cohort study, which will likely boost its scientific impact and value.

1. Significance:

Strengths

- This project combines a highly significant topic – the effects of stimulant use on HIV transmission risk – with a highly important key population focus – Black MSM – and novel methods approaches (in particular, views on networks and neighborhood factors) – to likely lead to ground-breaking findings.
- This R21 leverages an ongoing NIH-funded cohort study, the “Neighborhoods and Networks (N2) Cohort Study” (R01MH112406). The two PIs of the ongoing study, Profs. Duncan and Schneider, are sub-award PIs on this current proposal.
- The embeddedness of this research in a larger research initiative will likely boost the scientific impact and value of the proposed work.
- In particular, specific aim 2 is likely to generate ground-breaking insights into HIV transmission among Black MSM: contributions of neighborhood and network characteristics to transmission risk.
- The new Intervention Translation focus boosts the likely relevance and policy impact of this ground-breaking study.

Weaknesses

- While the findings are likely to be ground-breaking in general, the study may lack statistical power to support strong conclusions this weakness is mitigated through the use of qualitative methods to further support hypothesis generation.

2. Investigator(s):

Strengths

- Outstanding team of scientifically highly productive investigators.
- The investigators are highly qualified for this research and have a strong past track record in the thematic domain of stimulant use and the methods domain of network analyses.

Weaknesses

- None noted.

3. Innovation:

Strengths

- While social and sexual network studies have been written and talked about much, there is still a relative scarcity of strong empirical research, in particular among the important key populations of the HIV epidemic.
- The social and sexual network data elicitation is likely to lead to ground-breaking insights – in particular, because the investigators will leverage three different data sources for network information: survey self-report, Facebook and cell phone contacts.

Weaknesses

- None noted.

4. Approach:

Strengths

- Detailed description and referencing of the many important and rigorously measured outcomes that the parent study contributes to this proposal; the N2 Cohort Study, will provide data that will be used and complemented in this proposed work (Table 3).
- The longitudinal data density is relatively high (most measures are available at 5 time points).
- The additional measures that will be added through this work include a range of biomarkers – urine assays of stimulant use, DBS-based PrEP use and adherence screening, cytokines and chemokines to measure rectal inflammation.
- The survey-based social network measures are supplemented by two objective network data sources – Facebook and cell phone lists – which is a powerful data complement and provides opportunities for validation studies.
- It is a strength that the first-cycle coding approaches are already envisioned ex ante and named in detail and that the IDI results will be presented and discussed in the planned Intervention Translation Workshop.

Weaknesses

- The additional biomarker and behavioral data that this study will add to the N2 Cohort Study will only be measured in 40 cohort participants. This limitation is mitigated by the potential for this data collection to provide preliminary data for further funding application to expand measurement to the entire cohort.
- The social and sexual network data will be ego-centric – socio-centric network data is of course difficult to elicit but would massively boost the potential to advance our understanding of network effects.
- The key population that is the focus of this research may not be mainly on Facebook but use other social media – supporting evidence that Facebook is the best social media for this population would be useful (and/or other social media as additional data sources, such as Grinder, etc.).
- More machine-learning methods elements would provide further value – the information criterion model selection that is currently planned could be boosted, e.g., through cross-validation-based model selection.
- This project provides powerful opportunities for mixed methods research; but such integrating approaches do not seem to be envisioned.

5. Environment:

Strengths

- Excellent environment in NYC and in national collaborations.
- The involvement and close collaboration of the parent study PIs in this research is a strength.

Weaknesses

- None noted.

Study Timeline:

Strengths

- Not Applicable (No Clinical Trials)

Weaknesses

- Not Applicable (No Clinical Trials)

Protections for Human Subjects:

Acceptable Risks and/or Adequate Protections

- The study has strong human subjects protection plans and will leverage the rigorous and safe parent study.

Data and Safety Monitoring Plan (Applicable for Clinical Trials Only):

Not Applicable (No Clinical Trials)

Inclusion Plans:

- Sex/Gender: Distribution justified scientifically
- Race/Ethnicity: Distribution justified scientifically
- For NIH-Defined Phase III trials, Plans for valid design and analysis: Not applicable
- Inclusion/Exclusion Based on Age: Distribution justified scientifically
- The focus of this research is Black MSM.

Vertebrate Animals:

Not Applicable (No Vertebrate Animals)

Biohazards:

Not Applicable (No Biohazards)

Resubmission:

- This resubmission is highly responsive to the previous reviewers' comments. In particular, the investigators have substantially improved the description of the measures collected in the parent cohort study and added important methods details (such as how they will deal with network decay and how they will explore bio-behavioral mechanisms of the effects of stimulant use on HIV transmission).

- Another major improvement is the stronger focus on insight for intervention ideation and design. In particular, the research now includes reflections and feedback on promising novel and existing interventions and the team has added an Intervention Translation Workshop to facilitate the translation of study findings into interventions.
- The investigator team has further improved the integration of the different study components. An opportunity that still seems to be missed is a strong integration of specific aims 2 and 3 -- the effects of stimulant use on HIV transmission are likely strongly dependent on network characteristics, such as network turnover and mixing patterns, but specific aim 3 still focuses on individual-level factors (PrEP adherence, condom use, ART adherence, rectal inflammation).

Applications from Foreign Organizations:

Not Applicable (No Foreign Organizations)

Select Agents:

Not Applicable (No Select Agents)

Resource Sharing Plans:

Acceptable

- I could not find a data sharing plan; the reason may be that such sharing plans are not required for NIH funding <500,000.

Authentication of Key Biological and/or Chemical Resources:

Not Applicable (No Relevant Resources)

Budget and Period of Support:

Recommend as Requested:

THE FOLLOWING SECTIONS WERE PREPARED BY THE SCIENTIFIC REVIEW OFFICER TO SUMMARIZE THE OUTCOME OF DISCUSSIONS OF THE REVIEW COMMITTEE, OR REVIEWERS' WRITTEN CRITIQUES, ON THE FOLLOWING ISSUES:

PROTECTION OF HUMAN SUBJECTS: ACCEPTABLE

INCLUSION OF WOMEN PLAN: ACCEPTABLE

INCLUSION OF MINORITIES PLAN: ACCEPTABLE

INCLUSION ACROSS THE LIFESPAN: ACCEPTABLE

COMMITTEE BUDGET RECOMMENDATIONS: The budget was recommended as requested.

Footnotes for 1 R21 DA053156-01A1; PI Name: Knox, Justin

+ Derived from the range of percentile values calculated for the study section that reviewed this application.

NIH has modified its policy regarding the receipt of resubmissions (amended applications).See Guide Notice NOT-OD-18-197 at https://grants.nih.gov/grants/guide/notice-files/NOT-OD-18-197.html. The impact/priority score is calculated after discussion of an application by averaging the overall scores (1-9) given by all voting reviewers on the committee and multiplying by 10. The criterion scores are submitted prior to the meeting by the individual reviewers assigned to an application, and are not discussed specifically at the review meeting or calculated into the overall impact score. Some applications also receive a percentile ranking. For details on the review process, see http://grants.nih.gov/grants/peer_review_process.htm#scoring.

MEETING ROSTER

Population and Public Health Approaches to HIV/AIDS Study Section
Healthcare Delivery and Methodologies Integrated Review Group
CENTER FOR SCIENTIFIC REVIEW
PPAH
03/11/2021 - 03/12/2021

Notice of NIH Policy to All Applicants: Meeting rosters are provided for information purposes only. Applicant investigators and institutional officials must not communicate directly with study section members about an application before or after the review. Failure to observe this policy will create a serious breach of integrity in the peer review process, and may lead to actions outlined in NOT-OD-14-073 at https://grants.nih.gov/grants/guide/notice-files/NOT-OD-14-073.html and NOT-OD-15-106 at https://grants.nih.gov/grants/guide/notice-files/NOT-OD-15-106.html, including removal of the application from immediate review.

CHAIRPERSON(S)

BAUERMEISTER, JOSE ARTURO, PHD
PROFESSOR
DEPARTMENT OF FAMILY AND COMMUNITY HEALTH
SCHOOL OF NURSING
UNIVERSITY OF PENNSYLVANIA
PHILADELPHIA, PA 19104

MEMBERS

BARNIGHAUSEN, TILL, MD
PROFESSOR AND DIRECTOR
HEIDELBERG INSTITUTE OF GLOBAL HEALTH
FACULTY OF MEDICINE AND UNIVERSITY HOSPITAL
UNIVERSITY OF HEIDELBERG
HEIDELBERG 69120
GERMANY

BAUMAN, LAURIE J, PHD
PROFESSOR
DEPARTMENT OF PEDIATRICS
ALBERT EINSTEIN COLLEGE OF MEDICINE
BRONX, NY 10461

BIRKETT, MICHELLE, PHD
ASSISTANT PROFESSOR
DEPARTMENT OF MEDICAL SOCIAL SCIENCES
AND PREVENTIVE MEDICINE
FEINBERG SCHOOL OF MEDICINE
NORTHWESTERN UNIVERSITY
CHICAGO, IL 60611

BRAITSTEIN, PAULA KARINA ALICE, PHD
ASSOCIATE PROFESSOR
DEPARTMENT OF OBSTETRICS AND GYNECOLOGY
DALLA LANA SCHOOL OF PUBLIC HEALTH
UNIVERSITY OF TORONTO
TORONTO, ON M5T 3M7
CANADA

BROWNE, FELICIA AMIRA, BS, MPH, SCD *
SENIOR RESEARCH PUBLIC HEALTH ANALYST
RTI INTERNATIONAL
SUBSTANCE USE, GENDER AND APPLIED RESEARCH PROGRAM
RESEARCH TRIANGLE PARK, NC 27709

BUI, THANH C, MPH, MD, DPH *
ASSISTANT PROFESSOR
HEALTH PROMOTION RESEARCH CENTER
UNIVERSITY OF OKLAHOMA
OKLAHOMA CITY, OK 73104

CARNEGIE, NICOLE BOHME, BA, MS, PHD *
ASSOCIATE PROFESSOR
DEPARTMENT OF STATISTICS
MONTANA STATE UNIVERSITY
BOZEMAN, MT 59715

CHAMPION, JANE DIMMITT, PHD, BS, MS, MA, DNP *
PROFESSOR
SCHOOL OF NURSING
UNIVERSITY OF TEXAS AT AUSTIN
AUSTIN, TX 78701

DARBES, LYNAE A, PHD
ASSOCIATE PROFESSOR
DEPARTMENT OF HEALTH BEHAVIOR
AND BIOLOGICAL SCIENCES
SCHOOL OF NURSING
UNIVERSITY OF MICHIGAN
ANN ARBOR, MI 48109

FEASTER, DANIEL J, PHD, BA, MS *
PROFESSOR
DIVISION OF BIOSTATISTICS
DEPARTMENT OF PUBLIC HEALTH SCIENCES
MILLER SCHOOL OF MEDICINE
UNIVERSITY OF MIAMI
MIAMI, FL 33136

FONG, YOUYI, PHD *
PROFESSOR
VACCINE AND INFECTIOUS DISEASE DIVISION
PUBLIC HEALTH SCIENCES DIVISION
FRED HUTCHINSON CANCER RESEARCH CENTER
SEATTLE, WA 98109-1024

FUJIMOTO, KAYO, PHD
DISTINGUISHED PROFESSOR
DEPARTMENT OF HEALTH PROMOTION
AND BEHAVIORAL SCIENCES
SCHOOL OF PUBLIC HEALTH
UNIVERSITY OF TEXAS HEALTH SCIENCE CENTER
HOUSTON, TX 77030

GENG, ELVIN H., MD, MPH *
PROFESSOR
DIVISION OF INFECTIOUS DISEASES
DEPARTMENT OF INTERNAL MEDICINE
WASHINGTON UNIVERSITY
ST. LOUIS, MO 63110

HABERER, JESSICA ELIZABETH, MD
ASSOCIATE PROFESSOR
DEPARTMENT OF MEDICINE
MASSACHUSETTS GENERAL HOSPITAL
BOSTON, MA 02114

HOGAN, JOSEPH W, BS, MS, SCD *
CAROLE AND LAWRENCE SIROVICH PROFESSOR OF
PUBLIC HEALTH
DEPARTMENT OF BIOSTATISTICS
SCHOOL OF PUBLIC HEALTH
BROWN UNIVERSITY
PROVIDENCE, RI 02912

KERSHAW, TRACE S, PHD
PROFESSOR
DEPARTMENT OF EPIDEMIOLOGY
CENTER FOR INTERDISCIPLINARY RESEARCH ON AIDS
SCHOOL OF PUBLIC HEALTH
YALE UNIVERSITY
NEW HAVEN, CT 06510

KIENE, SUSAN MARIA, PHD *
PROFESSOR
DIVISION OF EPIDEMIOLOGY AND BIOSTATISTICS
SCHOOL OF PUBLIC HEALTH
SAN DIEGO STATE UNIVERSITY
SAN DIEGO, CA 92182

LI, JIANGHONG, MD, MS, MPH *
SENIOR RESEARCH ASSOCIATE
INSTITUTE FOR COMMUNITY RESEARCH
CENTER FOR INTERDISCIPLINARY RESEARCH ON AIDS
HARTFORD, CT 06106

MARHEFKA, STEPHANIE LYNN, BPHL, MS, PHD *
PROFESSOR AND ASSISTANT DEAN FOR RESEARCH
COLLEGE OF PUBLIC HEALTH
UNIVERSITY OF SOUTH FLORIDA
TAMPA, FL 33620

MCCLELLAND, RAYMOND SCOTT, MD
PROFESSOR
DEPARTMENTS OF MEDICINE, EPIDEMIOLOGY,
AND GLOBAL HEALTH
SCHOOL OF MEDICINE
UNIVERSITY OF WASHINGTON
SEATTLE, WA 98104

MCMAHON, JAMES M, PHD
ASSOCIATE PROFESSOR
SCHOOL OF NURSING
UNIVERSITY OF ROCHESTER MEDICAL CENTER
ROCHESTER, NY 14642

MEEK, ERIN, PHD
SENIOR RESEARCH SCIENTIST
AIDS OFFICE
SAN FRANCISCO DEPARTMENT OF PUBLIC HEALTH
SAN FRANCISCO, CA 94102

MOODY, JAMES, PHD, MA, BS *
PROFESSOR
DEPARTMENT OF SOCIOLOGY
TRINITY COLLEGE OF ARTS & SCIENCES
DUKE UNIVERSITY
DURHAM, NC 27708

NAAR, SYLVIE, PHD *
PROFESSOR
DEPARTMENT OF BEHAVIORAL SCIENCES
AND SOCIAL MEDICINE
FLORIDA STATE UNIVERSITY
TALLAHASSEE, FL 32304

PAGE, KIMBERLY, PHD
PROFESSOR
DIVISION OF EPIDEMIOLOGY, BIOSTATISTICS,
AND PREVENTIVE MEDICINE
DEPARTMENT OF INTERNAL MEDICINE
UNIVERSITY OF NEW MEXICO HEALTH SCIENCES CENTER
ALBUQUERQUE, NM 87131

PHO, MAI TUYET, MD, MPH *
ASSISTANT PROFESSOR
DEPARTMENT OF MEDICINE
SECTION OF INFECTIOUS DISEASES AND GLOBAL HEALTH
UNIVERSITY OF CHICAGO MEDICAL CENTER
CHICAGO, IL 60637

SALEMI, MARCO, PHD
PROFESSOR
DEPARTMENT OF PATHOLOGY, IMMUNOLOGY,
AND LABORATORY MEDICINE
COLLEGE OF MEDICINE
UNIVERSITY OF FLORIDA
GAINESVILLE, FL 32610

SCHNALL, REBECCA, MBA, MPH, PHD, RN *
MARY DICKEY LINDSAY ASSOCIATE PROFESSOR
HEALTH PROMOTION AND DISEASE PREVENTION
SCHOOL OF NURSING
COLUMBIA UNIVERSITY
NEW YORK, NY 10032

SHEPHERD, BRYAN EARL, PHD *
PROFESSOR AND VICE CHAIR
DEPARTMENT OF BIOSTATISTICS
SCHOOL OF MEDICINE
VANDERBILT UNIVERSITY
NASHVILLE, TN 37232-2158

SHERMAN, SUSAN GAIL, PHD
PROFESSOR
DEPARTMENT OF HEALTH, BEHAVIOR, AND SOCIETY
SCHOOL OF PUBLIC HEALTH
JOHNS HOPKINS UNIVERSITY
BALTIMORE, MD 21205

STORY, WILLIAM THOMAS, BA, MPH, PHD *
ASSISTANT PROFESSOR
COLLEGE OF PUBLIC HEALTH
UNIVERSITY OF IOWA
IOWA CITY, IA 52242

SWEAT, MICHAEL D, PHD
PROFESSOR
DEPARTMENT OF PSYCHIATRY
AND BEHAVIORAL SCIENCES
MEDICAL UNIVERSITY OF SOUTH CAROLINA
CHARLESTON, SC 29407

WALDROP, DRENNA, PHD *
PROFESSOR
DEPARTMENT OF NURSING
NELL HODGSON WOODRUFF SCHOOL OF NURSING
EMORY UNIVERSITY
ATLANTA, GA 30322

YANG, XIUSHI, BS, MA, PHD *
PROFESSOR
DEPARTMENT OF SOCIOLOGY AND CRIMINAL JUSTICE
OLD DOMINION UNIVERSITY
NORFOLK, VA 23529

YOUNG, APRIL MARIE, MPH, PHD
ASSOCIATE PROFESSOR
DEPARTMENT OF EPIDEMIOLOGY
COLLEGE OF PUBLIC HEALTH
UNIVERSITY OF KENTUCKY
LEXINGTON, KY 40536

MAIL REVIEWER(S)

CHENG, DEBBIE M., BS, SCD
PROFESSOR
DEPARTMENT OF BIOSTATISTICS
SCHOOL OF PUBLIC HEALTH
BOSTON UNIVERSITY
BOSTON, MA 02118

LOUE, SANA, JD, MPH, PHD
PROFESSOR AND VICE-DEAN
CENTER FOR MINORITY HEALTH
DEPARTMENT OF EPIDEMIOLOGY AND BIOSTATISTICS
SCHOOL OF MEDICINE
CASE WESTERN RESERVE UNIVERSITY
CLEVELAND, OH 44106

SCIENTIFIC REVIEW OFFICER

GUERRIER, JOSE H, PHD
SCIENTIFIC REVIEW OFFICER
CENTER FOR SCIENTIFIC REVIEW
NATIONAL INSTITUTES OF HEALTH
BETHESDA, MD 20892

EXTRAMURAL SUPPORT ASSISTANT

NJOKU, PHILIP C
EXTRAMURAL SUPPORT ASSISTANT
DIVISION OF AIDS, BEHAVORIAL, POPULATION SCIENCES
HEALTHCARE DELIVERY AND METHODOLOGIES (HDM)
NATIONAL INSTITUTES OF HEALTH
BETHESDA, MD 20892

* Temporary Member. For grant applications, temporary members may participate in the entire meeting or may review only selected applications as needed.

Consultants are required to absent themselves from the room during the review of any application if their presence would constitute or appear to constitute a conflict of interest.

SUMMARY STATEMENT
(Privileged Communication)

PROGRAM CONTACT:
SHILPY Dixit

Release Date: **05/06/2021**
Revised Date:

Application Number: **1 R01 HL160325-01**

Principal Investigators (Listed Alphabetically):

DUNCAN, DUSTIN T (Contact)
SCHNEIDER, JOHN

Applicant Organization: COLUMBIA UNIVERSITY HEALTH SCIENCES

Review Group: **ZHL1 CSR-Q (M1)**
National Heart, Lung, and Blood Institute Special Emphasis Panel
Using Syndemics to Understand HLBS Disease in People with HIV AIDS

Meeting Date: **04/16/2021**
Council: **MAY 2021**
Requested Start: **07/01/2021**
RFA/PA: **HL21-018**
PCC: **SLSM A**

Project Title: **Characterizing Sleep, ART Adherence and Viral Suppression Among Black Sexual Minority Men**
SRG Action: **Impact Score:32**
Next Steps: **Visit https://grants.nih.gov/grants/next_steps.htm**
Human Subjects: 30-Human subjects involved - Certified, no SRG concerns
Animal Subjects: 10-No live vertebrate animals involved for competing appl.
Gender: 3A-Only men, scientifically acceptable
Minority: 2A-Only minorities, scientifically acceptable
Age: 6A-Children and Adults, scientifically acceptable

Project Year	Direct Costs Requested	Estimated Total Cost
1	483,375	793,118
2	484,735	795,350
3	484,687	795,271
4	484,524	795,003
5	484,908	795,633
TOTAL	2,422,229	3,974,375

ADMINISTRATIVE BUDGET NOTE: The budget shown is the requested budget and has not been adjusted to reflect any recommendations made by reviewers. If an award is planned, the costs will be calculated by Institute grants management staff based on the recommendations outlined below in the COMMITTEE BUDGET RECOMMENDATIONS section.

1R01HL160325-01 Duncan, Dustin

RESUME AND SUMMARY OF DISCUSSION: Drs. Duncan and Schneider have submitted a new application entitled, “Characterizing Sleep, ART Adherence and Viral Suppression Among Black Sexual Minority Men,” in response to RFA-HL-21-018, Using Syndemics to Understand HLBS Disease Emergence and Progression in People with HIV (PWH). The review panel identified many strengths in this application led by multiple PIs with complementary expertise and a history of collaboration. The team of investigators is well-qualified to carry out the study that includes syndemic approaches. The focus on sleep is important and innovative and the target population is of high significance. The available study cohort is an additional strength. Some weaknesses were also identified which moderated enthusiasm, including insufficient justification of the obesity aim, and some potential missed opportunities to gather additional data. However, reviewers felt that the strengths of the application outweighed the weaknesses and that the project could have a potentially high impact. Overall, the review panel rated this application as excellent.

DESCRIPTION (provided by applicant): To address the aims of the proposed research and RFA-HL-21-018, we will use a syndemics and multi-level approach to investigate relationships between sleep and HIV treatment outcomes and behaviors (e.g., viral suppression and retention in care) cross-sectionally and longitudinally among Black gay, bisexual and other sexual minority men (SMM) followed over one year to inform interventions. We will enroll 250 Black SMM from the NIH-funded Neighborhoods and Networks (N2) Cohort Study in the proposed N2 Sleep Health Study to address the aims of the research. Eligibility requirements include: HIV-seropositive and self-reported willingness to wear a wrist actigraph for two-weeks at three points over the course of a year. In this longitudinal study, after completing the initial 2-week wrist actigraphy protocol, participants will carry the wrist actigraph for an additional 2-weeks every six-months over the one-year study period—for a total of three times. Objectively measured sleep data at baseline could potentially influence decision-making regarding HIV treatment (e.g., antiretroviral treatment [ART] outcomes) over time, providing a clear temporal ordering and an ability to consider potential time-lags. Multi-level factors – e.g., individual-level obesity, intimate partner violence, and spatial proximity to healthcare services – may modify these relationships. The proposed study will be the first objective sleep health study among any population of Black SMM. Findings from the proposed research have significant implications for targeting contextually appropriate sleep and HIV interventions as there is a need for new approaches to inform the next generation of HIV interventions (i.e., long-acting injectables), especially for Black SMM.

PUBLIC HEALTH RELEVANCE: Using a syndemic and multi-level approach, this project seeks to investigate relationships between sleep and HIV treatment outcomes and behaviors (e.g., viral suppression and retention in care) among Black gay, bisexual and other sexual minority men (SMM), a population in the United States heavily impacted by HIV/AIDS.

(End of abstract)

CRITIQUE 1:

Significance: 2
Investigator(s): 3
Innovation: 2
Approach: 4
Environment: 2

Overall Impact: This is very strong application which directly measures the influence of sleep quality on HIV outcomes among Black sexual minority men. The rationale is laid out very clearly and the purpose and potential influence on individual and population health of an eventual successful

intervention is profound. The Principal Investigators are highly qualified with a history of collaboration and they have assembled an experienced and talented group of co-investigators. Weaknesses noted are the lack of a specific investigator to interpret the sleep actigraphy data and a general lack of detail about how these important data will be interpreted. The obesity aim is not well justified and there are no preliminary data on the prevalence of obesity in the existing cohort to imply that aim hypothesis can be tested in this cohort.

1. Significance:
Strengths
- Addresses an important demographic, Black sexual minority men, who suffer from higher rates of HIV, poor health outcomes from HIV and other comorbidities, as well as syndemic mental health, substance abuse, poverty and violence.
- Sleep is profoundly important aspect of overall health and a modifiable one.
- Very important to improve HIV outcomes in this population both to improve individual health but also to prevent ongoing HIV transmission in the community.

Weaknesses
- It is unclear why obesity is singled out as a factor influencing the relationship between sleep and HIV outcomes, little data are provided about the prevalence of obesity in the existing N2 cohort or among the target populations. Nor is there justification for the hypothesis that obesity moderates this relationship.

2. Investigator(s):
Strengths
- PIs are well-qualified with complementary expertise, strong epidemiology, HIV expertise. The supporting team is well qualified to explore the syndemic stressors of Black SMM.

Weaknesses
- No investigator (though there are Advisory Committee members) with expertise in medical sleep science or with obvious experience with the interpretation of actigraphy/sleep diary data. No budgeted FTE to interpret the sleep data.

3. Innovation:
Strengths
- Sleep as a predictor of HIV outcomes has not been explored in this depth or this population.
- The candidate syndemic cluster is well studied but not in sleep health.

Weaknesses
- None noted.

4. Approach:
Strengths
- Longitudinal design will allow some assessment of causality.

Weaknesses
- No rationale is given for the timing and number of the sleep evaluations (why 3 evaluations and why q 6 months?), more information may be gleaned from a two year rather than a one year study, particularly with regard to the outcome of visit adherence but also viral suppression. Many PLWH only have 2 scheduled visits a year so the denominator for visit adherence may be lower than ideal to discriminate between levels of adherence. No rationale is given for 14 day actigraphy vs the more standard 7-10 day measurement.

- Influence of obesity is a primary aim but there are no data on the prevalence of obesity in the target population- do the investigators know that obesity is prevalent enough to test this hypothesis?
- Sleep apnea is mentioned as a potential outcome but there is no method mentioned for measuring sleep apnea. Measurement of sleep apnea may be beyond the scope of the project but would strengthen the approach to aim 3.
- Methodology for reviewing the actigraph and sleep diary data are not presented. Will an automated read of the actigraphy data be used, will any of the data be reviewed manually for validation, how will the sleep diaries be used? Circadian disruption is mentioned as a gap in the literature but no methodology for quantifying Circadian disruption is mentioned.
- Minor weakness/query- assessing the viral load at the post-actigraphy visit (rather than pre-actigraphy) may bias toward viral suppression as the participant is reminded frequently of the study by the actiwatch and the diary- it is possible that this may influence medication adherence during the 2 week period.

5. Environment:
Strengths
- University of Chicago and Columbia are strong research environments and provide the necessary resources for the success of the project.
- The existing cohort strengthens the feasibility of the application.

Weaknesses
- Unclear if the cohort represents a broad range of providers, (the investigators have access to the EHR of participants which implies they may be recruited from one of the investigators' clinical site(s)), This is a minor weakness as it is assumed they will recruit participants from a range of care sites but the investigators should articulate how they will make sure that differences in adherence and suppression are due to the participant/social environmental factors they are studying rather than the quality of care and outreach at a particular care site in Chicago.

Protections for Human Subjects:
Acceptable Risks and/or Adequate Protections

Data and Safety Monitoring Plan (Applicable for Clinical Trials Only):
Not Applicable (No Clinical Trials)

Inclusion Plans:
- Sex/Gender: Distribution justified scientifically
- Race/Ethnicity: Distribution justified scientifically
- For NIH-Defined Phase III trials, Plans for valid design and analysis: Not applicable
- Inclusion/Exclusion Based on Age: Distribution justified scientifically

Vertebrate Animals:
Not Applicable (No Vertebrate Animals)

Biohazards:
Not Applicable (No Biohazards)

Applications from Foreign Organizations:
Not Applicable (No Foreign Organizations)

Select Agents:
Not Applicable (No Select Agents)

Resource Sharing Plans:
Acceptable

Authentication of Key Biological and/or Chemical Resources:
Not Applicable (No Relevant Resources)

CRITIQUE 2:

Significance: 3
Investigator(s): 2
Innovation: 3
Approach: 2
Environment: 1

Overall Impact: The researchers propose to study the relationship of sleep with HIV care outcomes, including viral suppression and retention in care, among young Black sexual minority men using a syndemics-informed and multi-level analysis that also incorporates obesity and individual-level variables commonly associated with syndemics and HIV. The team includes a long-standing collaboration of accomplished researchers with background and skills for each of the areas of concern. The proposal is significant and innovative in that it uses objective measures of the primary outcomes of interest and explores the problems of sleep and obesity among the US's population hardest hit by HIV/AIDS. If successful, the findings could inform clinical practice and strengthen efforts to reduce the burden of HIV. The research plan is well described generally, but it is less clear how traditional syndemic factors associated with HIV studies are measured and included. Further, the inclusion of obesity is important as a factor in and of itself, but it is less clear if/how obesity is directly related to the HIV outcomes or through its relationship with sleep.

1. Significance:
Strengths
- HIV affects young Black SMM in a highly disproportionate way. Enhancing treatment and reducing infections remains highly important. This study proposes to examine how sleep and HIV treatment affect one another. Objectively measuring sleep among Black SMM and determining its relationship with medication adherence.
- Obesity is also a major national epidemic. Understanding its relationship to both sleep and HIV is needed and determining if obesity is a moderator may be useful.
- Exploring how sleep may influence mental health and stress as a pathway to HIV transmission behaviors is very interesting.
- Advancing the use of an objective measure of sleep in an understudied population is a strength.

Weaknesses
- The proposal did not clearly identify the epidemiology of obesity, particularly as related to the proposed sample of young Black SMM. It is unclear how the researchers believe obesity is related to the HIV outcomes.
- The aims do not include a focus on sexual behavior, but the investigators have the ability to explore syndemic influences on it.

2. Investigator(s):

Strengths
- The team has a strong history of working well together.
- Each member of the team has complementary strengths that span HIV, sleep, studies of Black SMM, etc. They also bring a range of experiences and talents in complementary methods.

Weaknesses
- There is not an obesity expert.

3. Innovation
Strengths
- The study incorporates technologies for increasing objectivity in sleep and uses current technologies for obesity and HIV viral load.
- The study proposes both syndemic and multi-level approaches.
- Better understanding the health of young Black SMM advances an intersectional minority research agenda.

Weaknesses
- Studying Black SMM is clearly important and focusing solely on this population is important rather than comparing across racial/ethnic identities.
- A variety of factors traditionally included in studying syndemics among sexual minorities are included (IPV, drinking, drug use), but it is unclear how investigators see these related to cognition and mental health in their conceptual model. Similarly, it is unclear how experiences of racism, antigay bias and HIV stigma are theorized.

4. Approach
Strengths
- The research plan is clearly articulated and seems doable in the timeframe allocated.
- The investigators have ready access to the study population and have demonstrated good retention in prior studies. They describe a strategy for achieving the sample if the parent study does not yield sufficient enrollment.
- Objective measures of the primary variables are included: sleep, viral load, retention in care, and obesity.

Weaknesses
- Eligibility is stated as 16-34 in one place and 18-35 elsewhere. Perhaps the former is for the N2 Sleep Study and the latter is for this proposed study?
- It is unclear how the sleep diary is kept by participants. Is it digital or paper?
- There is scant description of the syndemic and multi-level variables.
- It is unclear if the investigators are including measures of experiences of racism, antigay bias and HIV stigma.
- The investigators include sexual behavior outcomes, but these are not described as aims.

5. Environment:
Strengths
- Each partner institution is very strong.
- The inclusion of the longstanding projects in Chicago and collaborative arrangements in Chicago are very good.

Weaknesses
- None noted.

Study Timeline:
Strengths
- The proposed research seems doable in the time allocated.

Weaknesses
- None.

Protections for Human Subjects
Acceptable Risks and/or Adequate Protections
- Existing relationships with the study population and community are established.

Data and Safety Monitoring Plan (Applicable for Clinical Trials Only):
Not Applicable (No Clinical Trials)

Inclusion Plans:
- Sex/Gender: Distribution justified scientifically
- Race/Ethnicity: Distribution justified scientifically
- Inclusion/Exclusion Based on Age: Distribution justified scientifically
- Focusing solely on young Black SMM is acceptable.

Vertebrate Animals:
Not Applicable (No Vertebrate Animals)

Resource Sharing Plans:
Acceptable

Authentication of Key Biological and/or Chemical Resources:
Not Applicable (No Relevant Resources)

Budget and Period of Support:
Recommend as Requested

CRITIQUE 3:

Significance: 1
Investigator(s): 1
Innovation: 1
Approach: 2
Environment: 1

This is a truly excellent study: the researchers are strong, the methodology is robust, and the plans are reasonable (especially building upon an existing cohort). The only real hesitation this reviewer has about the study is that the only “qualitative” data will come in year 5 to evaluate whether the team’s investigative findings were salient with the study population. This is a missed opportunity, especially because many applicants on the research team have experience working in both qualitative and quantitative approaches. Although the theory is clear and the methods for taking stress/social dimensions seriously are well thought out, without some ethnographic component (even 30 in depth interviews), some of this deeper knowledge will be lost. This reviewer is enthusiastic about this project, but suggests that the team bring on an ethnographer—hopefully one with deep ties to the Chicago area who has worked with Black SSM or in relation to the preexisting cohort to study how people think and feel about sleep and the factors that drive good or poor sleep. Either 30 in depth interviews with participants, or 10 elongated ethnographic observations would be ideal to capture a deeper and more robust understanding of the syndemic features the team will capture with their larger dataset of 250.

(End of Reviewer’s Comments)

THE FOLLOWING SECTIONS WERE PREPARED BY THE SCIENTIFIC REVIEW OFFICER TO SUMMARIZE THE OUTCOME OF DISCUSSIONS OF THE REVIEW COMMITTEE, OR REVIEWERS' WRITTEN CRITIQUES, ON THE FOLLOWING ISSUES:

VERTEBRATE ANIMALS: NOT APPLICABLE

PROTECTION OF HUMAN SUBJECTS: ACCEPTABLE

INCLUSION OF WOMEN PLAN: ACCEPTABLE

INCLUSION OF MINORITIES PLAN: ACCEPTABLE

INCLUSION ACROSS THE LIFESPAN PLAN: ACCEPTABLE

RESOURCE SHARING PLANS: ACCEPTABLE

COMMITTEE BUDGET RECOMMENDATIONS: The budget was recommended as requested.

Footnotes for 1 R01 HL160325-01; PI Name: Duncan, Dustin T

NIH has modified its policy regarding the receipt of resubmissions (amended applications).See Guide Notice NOT-OD-18-197 at https://grants.nih.gov/grants/guide/notice-files/NOT-OD-18-197.html. The impact/priority score is calculated after discussion of an application by averaging the overall scores (1-9) given by all voting reviewers on the committee and multiplying by 10. The criterion scores are submitted prior to the meeting by the individual reviewers assigned to an application, and are not discussed specifically at the review meeting or calculated into the overall impact score. Some applications also receive a percentile ranking. For details on the review process, see http://grants.nih.gov/grants/peer_review_process.htm#scoring.

MEETING ROSTER

The roster for this review meeting is displayed as an aggregated roster that includes reviewers from multiple HL Special Emphasis Panels of the NHLBI-2 for the 2021/05 council round.
This roster for HL is available at:
http://public.era.nih.gov/pubroster/Reports?DOCTYPE=SEP&DESFORMAT=PDF&AGENDA_SEQ_NUM_P=414793
